# Endophytic Life Strategies Decoded by Genome and Transcriptome Analyses of the Mutualistic Root Symbiont *Piriformospora indica*

**DOI:** 10.1371/journal.ppat.1002290

**Published:** 2011-10-13

**Authors:** Alga Zuccaro, Urs Lahrmann, Ulrich Güldener, Gregor Langen, Stefanie Pfiffi, Dagmar Biedenkopf, Philip Wong, Birgit Samans, Carolin Grimm, Magdalena Basiewicz, Claude Murat, Francis Martin, Karl-Heinz Kogel

**Affiliations:** 1 Department of Organismic Interactions, Max-Planck Institute (MPI) for Terrestrial Microbiology, Marburg, Germany; 2 Institute of Bioinformatics and Systems Biology, Helmholtz Zentrum München (GmbH), Neuherberg, Germany; 3 Institute of Phytopathology and Applied Zoology, Research Centre for BioSystems, LandUse, and Nutrition (IFZ), Justus Liebig University, Giessen, Germany; 4 Biometry and Population Genetics, Research Centre for BioSystems, LandUse, and Nutrition (IFZ), Justus Liebig University, Giessen, Germany; 5 INRA, UMR 1136, INRA-Nancy Université, Interactions Arbres/Microorganismes, Champenoux, France; University of Melbourne, Australia

## Abstract

Recent sequencing projects have provided deep insight into fungal lifestyle-associated genomic adaptations. Here we report on the 25 Mb genome of the mutualistic root symbiont *Piriformospora indica* (Sebacinales, Basidiomycota) and provide a global characterization of fungal transcriptional responses associated with the colonization of living and dead barley roots. Extensive comparative analysis of the *P. indica* genome with other Basidiomycota and Ascomycota fungi that have diverse lifestyle strategies identified features typically associated with both, biotrophism and saprotrophism. The tightly controlled expression of the lifestyle-associated gene sets during the onset of the symbiosis, revealed by microarray analysis, argues for a biphasic root colonization strategy of *P. indica*. This is supported by a cytological study that shows an early biotrophic growth followed by a cell death-associated phase. About 10% of the fungal genes induced during the biotrophic colonization encoded putative small secreted proteins (SSP), including several lectin-like proteins and members of a *P. indica*-specific gene family (DELD) with a conserved novel seven-amino acids motif at the C-terminus. Similar to effectors found in other filamentous organisms, the occurrence of the DELDs correlated with the presence of transposable elements in gene-poor repeat-rich regions of the genome. This is the first in depth genomic study describing a mutualistic symbiont with a biphasic lifestyle. Our findings provide a significant advance in understanding development of biotrophic plant symbionts and suggest a series of incremental shifts along the continuum from saprotrophy towards biotrophy in the evolution of mycorrhizal association from decomposer fungi.

## Author Summary

Plant-associated fungi comprise a wide range of lifestyles, such as biotrophy, necrotrophy and hemibiotrophy. Biotrophic fungi require actively metabolizing plant tissues and avoid extensive damage while keeping their host alive. They include pathogenic as well as mutualistic forms. Necrotrophic fungi, which kill host cells in advance of their own growth and obtain nutrients from the dead cells, comprise only pathogenic forms. An intermediate category is represented by the hemibiotrophic fungi. These require living host cells during part of their life cycles, but switch to necrotrophy at later colonization stages with detrimental effects on host survival and fitness and have therefore so far been classified as pathogens. Our study reveals that the mutualistic symbiont *Piriformospora indica* possesses biotroph-associated genomic adaptations, such as lack of genes involved in nitrogen metabolism and is therefore predicted to suffer from some metabolic deficiencies. In line with biotrophy, *P. indica* has a limited potential for damage and destruction shared by symbiotic fungi and obligate biotrophic pathogens, i.e. absence of genes potentially involved in biosynthesis of toxic secondary metabolites and cyclic peptides. On the other hand, *P. indica* shares genomic traits with saprotrophic and hemibiotrophic phytopathogenic fungi, such as the presence of an expanded enzyme arsenal which is weakly expressed during the initial biotrophic phase. Cytological evidence for biotrophic growth followed by a cell death-associated phase that results in a mutually beneficial outcome, supports the idea that *P. indica* represents a missing link between decomposer fungi and obligate biotrophic mutualists.

## Introduction

Plants in natural ecosystems often display a high degree of colonization by endophytic fungi. Since these fungi colonize their hosts without causing visible disease symptoms, they have often been overlooked and little attention has been paid to their impacts on plant communities. Endophytes exhibit a broad range of lifestyles along the saprotrophy-biotrophy continuum, depending on the fitness benefits conferred to their host, secondary metabolites production and their colonization strategies [1], [2], [3], [4], [5]. The filamentous fungus *Piriformospora indica* belongs to the order Sebacinales which represents the earliest diverging branch of the Agaricomycetes and the most basal basidiomyceteous order with mycorrhizal abilities [1], [6], [7]. Taxa within this fungal group are either facultative or, as in the more derived species, obligate biotrophs. *P. indica*, which was originally isolated from soil of the Indian Thar desert [8] is the asexual model organism for experimental studies in the Sebacinales. *P. indica* displays an endophytic lifestyle and has the ability to colonize the roots of a wide range of mono- and dicotyledonous plants, including members of the Brassicaceae (e.g. *Arabidopsis thaliana*) which are known as non-host plants for ecto- and arbuscular mycorrhiza [9]. Plants colonized by *P. indica* display a wide range of beneficial effects including enhanced host growth and resistance to biotic and abiotic stresses [10], [11], [12], [13], promotion of adventitious root formation in cuttings [14] and enhanced nitrate and phosphate assimilation [15], [16]. *P. indica* extensively colonizes the differentiation and the root hair zones inter- and intracellularly, while it is rarely detectable in the elongation and meristematic zones [17]. This colonization pattern distinguishes it from ecto- and arbuscular mycorrhizal fungi, which either grow only intercellularly or colonize predominantly the deeper cortex layers of younger parts of the root [18]. An additional difference between mycorrhiza fungi and *P. indica* is its dependence on host cell death for successful colonization [17]. In barley, the host cell death related growth phase is associated with the down regulation of the endoplasmic reticulum membrane-localized cell death regulator BAX INHIBITOR-1 (BI-1). Consistent with this, transgenic barley plants that express the barley BAX INHIBITOR gene under a constitutive promoter, show increased cell viability and reduced colonization [17]. Recent studies revealed a complex interplay between the plant root and *P. indica*, involving suppression of microbe-associated molecular pattern (MAMP)-triggered root innate immunity, modulation of secondary metabolism (including plant hormone biosynthesis), induction of cell death, and elicitation of systemic resistance responses [19], [20], [21], [22], [23], [24]. However little information is available on the fungal genes and pathways involved in the establishment and maintenance of the symbiosis [15], [25]. In this study we report on the genome of *P. indica* and provide a global characterization of fungal transcriptional responses to colonization of dead and living root tissues. Data from recent sequencing projects have provided novel insights into genomic traits associated with various lifestyles in fungi, including ectomycorrhizal fungi [26], [27], [28], [29], [30]. Cytological investigation and comparative analysis of *P. indica* genomic traits and gene expression profiles revealed substantial differences in colonization strategies compared to known ectomycorrhizal fungi providing first insights into root endophytic life strategies in the Basidiomycota.

## Results and Discussion

### Barley root colonization by *P. indica*

**Figure 1:**
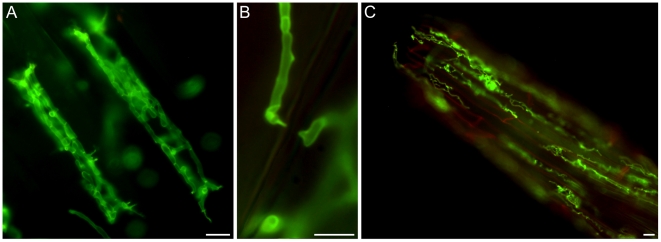
*P. indica* colonizing autoclaved barley root cells at 5 dpi. Fungal structures were stained with WGA-AF488 (green), plant cells were stained with propidium iodide (red). A) Cortex cells from autoclaved barley roots with fungal hyphae inside (5 dpi). B) Hyphal constriction at penetration site. C) Hyphae on and in the root tip. Images were taken with a Zeiss Axioplan fluorescence microscope with standard settings for WGA-AF488. The bars represent 10 µm.

**Figure 2:**
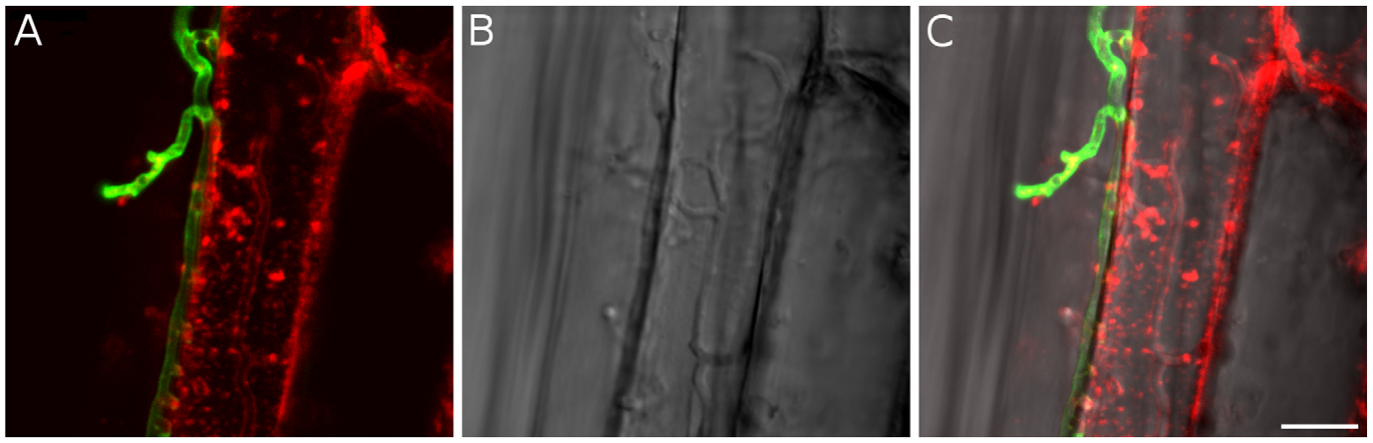
Biotrophic growth of *P. indica* in barley root cortex cells. Living root cortex cell with fungal hyphae inside (4 dpi). Fungal structures were stained with WGA-AF488 (green), plant membranes were stained with the endocytosis marker FM4-64 (red). A) FM4-64 and WGA-AF488, B) bright field, C) overlay. In contrast to extracellular hyphae, intracellular hyphae were not stainable with WGA-AF488, indicating that the hyphae remained enveloped in a plant-derived membrane throughout intracellular growth. Internalization of FM4-64 in the form of endomembrane structures after 20 min of incubation are visible inside the plant cell, indicating viability of the cells. Images were taken with a CLM, Leica TCS SP5 (Leica, Bensheim, Germany). The bar represents 10 µm.

**Figure 3:**
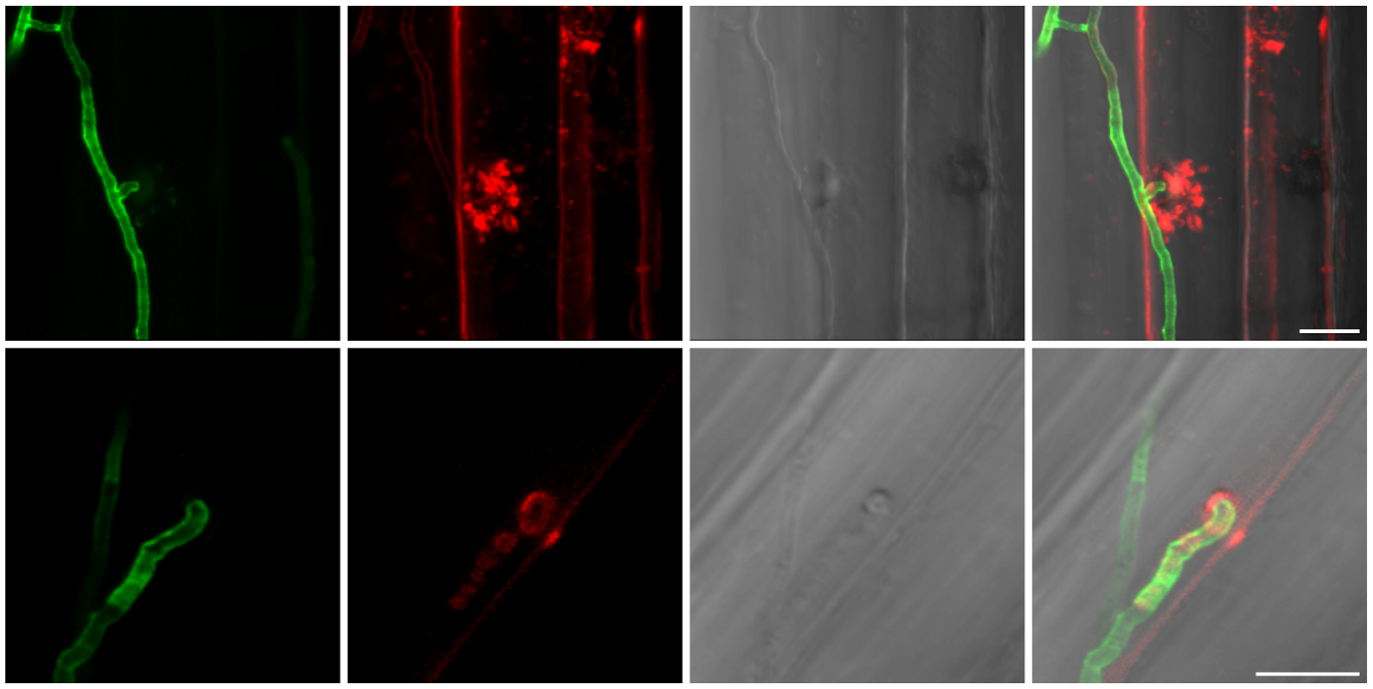
Penetration of hyphae in living root cortex cells. The upper panel shows vesicle transport at fungal penetration site. Fungal structures were stained with WGA-AF488 (green), plant membranes were stained with the endocytosis marker FM4-64 (red). The lower panel shows the presence of glycoproteins at the fungal penetration site. Fungal structures were stained with WGA-AF488 (green), α-mannopyranosyl and/or α-glucopyranosyl residues around the hyphal adhesion and penetration sites were stained with Concanavalin A (ConA-AF633, red) indicative of presence of glycoproteins. In barley leaves, papillae display a different composition from the adjacent cell wall and contain exosomes, H_2_O_2_, cell-wall cross linked proteins, thionins, callose, iron (Fe^3+^) and cell-wall cross linked phenolics but not cellulose and pectin [109], [110]. Less information is available on the papillae composition in barley root. From left to right: WGA-AF488 channel; FM4-64/ConA-AF633 channel; bright field; overlay. Images were taken with a CLM, Leica TCS SP5 (Leica, Bensheim, Germany). The bars represent 10 µm.

A detailed knowledge about the fungal colonization strategy is a prerequisite for the interpretation of transcriptome changes in response to endophytic root colonisation. To generate this information, roots from 3-day-old barley seedlings and autoclaved roots of the same age were inoculated with 500,000 chlamydospores/ml under sterile conditions and the colonization pattern was documented over a period of 7 days by fluorescence and confocal microscopy. Fungal growth in autoclaved barley roots, which retained their macroscopic structure and texture, was characterized by a massive intracellular development with highly branched hyphae from 3 days post inoculation (dpi) onwards (Figure 1). Newly produced chlamydospores were detected on the root surface at 5 dpi whereas intracellular chlamydospores were observed at 7 dpi. The early extensive intracellular hyphal development in dead cells resembled the colonization pattern of cells in living roots at later stages (>7 dpi), which prompted us to assess the viability of host cells during the symbiotic colonization. Colonized living roots were treated with both the fungal cell wall stain WGA-AF488 and the membrane stain FM4-64 that is commonly used for dissecting vesicles trafficking in living plant cells [31], [32], [33]. In agreement with a previous study [17], *P. indica* was confined to the cortex layer whereas the root tips and the central meristematic tissue were free of hyphae. Living cells, identified by the internalization of FM4-64 into endomembrane structures, were intracellularly colonized by a single hypha with no or limited branching from 3 dpi onwards (Figure 2). The failure of the WGA-AF488 to stain the hyphae inside living cells (Figure 2) strongly suggests that the fungus remained enveloped in an intact plant-derived membrane throughout intracellular growth. Formation of cell wall appositions (papillae) was observed sporadically during penetration attempts of living cortex cells. Presence of papillae, visualized with ConA-AF633 staining, correlated with the biotrophic phase of this fungus (Figure S1A). Closer inspection of the papillae showed accumulation of plant vesicles and glycoproteins at the penetration zone (Figure 3). These papillae were not always effective in stopping fungal penetration, indicating that *P. indica* is able to overcome plant cell wall-mediated defense in barley. At later colonization stages (>4 dpi) *P. indica* was more frequently detected in moribund or dead host cells which were extensively colonized by fungal hyphae. This cytological analysis revealed a mixture of colonized dead and living cells from 4 dpi onwards (Figure S1C and 1D).

### *P. indica* genome survey

Pyrosequencing of the *P. indica* genome was performed in parallel to RNA-Seq of cDNA pooled from different fungal developing stages. The genome was assembled into 1,884 scaffolds (size: >1 kb; N50: 51.83 kb) containing 2,359 contigs with an average read coverage of 22 and a genome size of 24.97 Mb. 11,769 gene models were identified using various *ab-initio* gene prediction programs and the open reading frames were validated by mapping unique expressed sequence tags (EST) to the scaffolds (Table S1). To assess the genome completeness of *P. indica* a blast search was performed with highly conserved core genes present in higher eukaryotes [34], [35]. From the expected 246 single-copy orthologs extracted from 21 genomes [35], 245 are present in the *P. indica* genome draft, indicating that >99% of the gene space is covered by the assembly. Protein blast searches (eVal: 10^−3^) showed that a large number of *P. indica's* predicted genes have closest matches for the ectomycorrhizal fungus *Laccaria bicolor* (3,109, 26.42%) and the saprotrophic fungus *Coprinopsis cinerea* (2,381, 20.23%), which therefore represent the closest related organisms sequenced at the present time. In addition a large number of genes have no orthologs in other genomes (3,286, 27.92%) (Figure S2). Synteny analysis showed only a minor number of conserved syntenic gene blocks between the genome of *P. indica* and those of *L. bicolor*, *C. cinerea* and *Ustilago maydis* (Figure S3). In comparison to the genome of related fungi *P. indica* has a significantly higher gene density with 471 ORFs/Mb (39% more ORFs/Mb than the average gene density of 338 ORFs/Mb calculated from 9 genomes, Table 1; S2), a low number of transposable elements (4.68%), and an absence of LTR gypsy elements in the repeat library, which are frequently found in other fungal genomes (Table S3). A specific identification of the reverse transcriptase 1 (RVT1) found in LTR gypsy confirmed that this elements are rare in the *P. indica* genome since only three RVT1 sequences were identified (data not shown). A relative abundance of 24 simple sequence repeats (SSRs)/Mb was identified in the *P. indica* draft genome which is in the lower range of fungal genomes. Additionally, with only 58 identified genomic tRNA genes *P. indica* has an unusual low number of these genes (Table S4). The codon usage preference of *P. indica* is comparable to that of other fungi (Figure S4).

**Table 1:** Main features of *P. indica* genome.

Genome size (Mb)	24.98
GC-content (%)	50.68
Repeat rate (%)	4.68
Protein coding genes	11,769
Average exons per gene	5.16
Gene density (number of genes per Mb)	471
Secreted proteins	867
Small secreted proteins (SSP)	386
Unique gene models	3,134
Unique SSP	197
tRNA genes	58

More details can be found in Table S2.

### Determination of ploidy

*P. indica* possesses multinucleate hyphae, but the failure to detect clamp connections or sexual reproduction has impaired the determination of ploidy [36]. We detected two allelic mating type loci with two genes encoding for homeodomain proteins in the *P. indica* genome (Figure S5). This finding is consistent either with a diploid nucleus or with a dikaryotic mycelium. To determine ploidy level, *P. indica* nuclei were stained with the DNA intercalating dye syto9 and fluorescence intensity (measured by CLSM) was compared to that of known DNA content from haploid and diploid forms of the reference organism *Saccharomyces cerevisiae*. The estimated DNA content of *P. indica* nuclei ranged from 15.3 to 21.3 Mb (Figure S6). This range is consistent with the genome size estimated by the pyrosequencing approach (Table 1), suggesting that *P. indica* nuclei are haploid. Single nucleotide polymorphisms (SNP) with two variants, were identified in about 92% of the contigs (23.15 Mb) with a frequency of 2.6 SNPs per kb and a total of 60,493 polymorphisms for the entire genome (Table S5). This value is similar to that observed in the diploid genome of *Candida albicans* [37]. Based on DNA content and SNP analysis, we conclude that *P. indica* is most likely a heterokaryon containing two genetically distinct nuclei. Average read coverage analysis of the contigs highlighted the presence of a group of genomic segments with half as many reads compared to the rest (Figure S7). A correlation between the occurrence of polymorphisms and sequence depth was found with no SNP observed for the contigs with an average read coverage of about 10 (Table S5 and Figure S8). These contigs probably represent highly polymorphic regions in the genome and account for 1.87 Mb sequence data with 1,056 predicted ORFs (Table S5 and Figure S8). The occurrence within these regions of the two highly syntenic contigs representing the two putative allelic mating type loci, which were not homologous enough to be assembled in one scaffold, further supports this conclusion (Figure S5).

### Domain and gene family expansion and contraction in the *P. indica* genome

To gain an overview of the biological processes and pathways that contribute to symbiosis, we compared the presence and abundance of individual protein functional regions in the *P. indica* predicted ORFs with the corresponding domain number in a broad range of fungal species using the Pfam database [38] (Table S6). The overall number of different domains represented in the *P. indica* genome (2,785) is comparable to that of other fungi (with an average value of 2,840 calculated from 10 genomes), but marked differences are present in terms of protein abundance per functional domain. Thirty-two protein domains are significantly expanded in the *P. indica* genome with fourteen of these exhibiting greater abundance than in any other genome analyzed in this study (Table S6). Expanded domains include proteins that are predicted to be involved in plant cell wall degradation (e.g., glycoside hydrolase families GH10, GH11 and GH61); proteolysis (e.g., metallopeptidases families M36 and M43); carbohydrate binding (e.g. protein containing LysM, WSC or CBM1 domains); protein binding (WD domain, G-beta repeat - WD40; NACHT domain; tetratricopeptide repeat - TPR_4 domain) together with proteins most probably involved in signaling and regulation of cellular responses to stress and nutrient availability (NB-ARC, G-alpha protein, F-box, RAS and RHO families) (Table S6 and S7). The expansion of protein binding motifs together with domains involved in signaling is evidence that *P. indica* owns a complex regulatory machinery that helps to sense and couple signals received from the external environment with the intracellular signaling pathways. These traits are shared by the ectomycorrhizal fungus *L. bicolor* but not by the saprotrophic fungus *C. cinerea* (Table S6), supporting the contention that some of these proteins are candidates for the regulation of a complex communication system between the mycobiont and its host [30]. In particular, the expansion of genes encoding NWD proteins, associating the NACHT and the WD-repeat domains (with 99 ORFs) in the *P. indica* genome (Table S6) is significant. WD-repeat proteins are found in all eukaryotes and coordinate multi-protein complex assemblies. Their combination with the NACHT NTPases, which share similarities in domain architecture with AP-ATPases, is found in a variety of proteins controlling programmed cell death, known as the incompatibility reaction, ensuring innate immunity in plants and animals towards microbial pathogens. It is therefore possible that this expansion of NWD genes might reflect the evolution of systems that function in non-self recognition and fungal innate immunity [39], [40].

**Figure 4:**
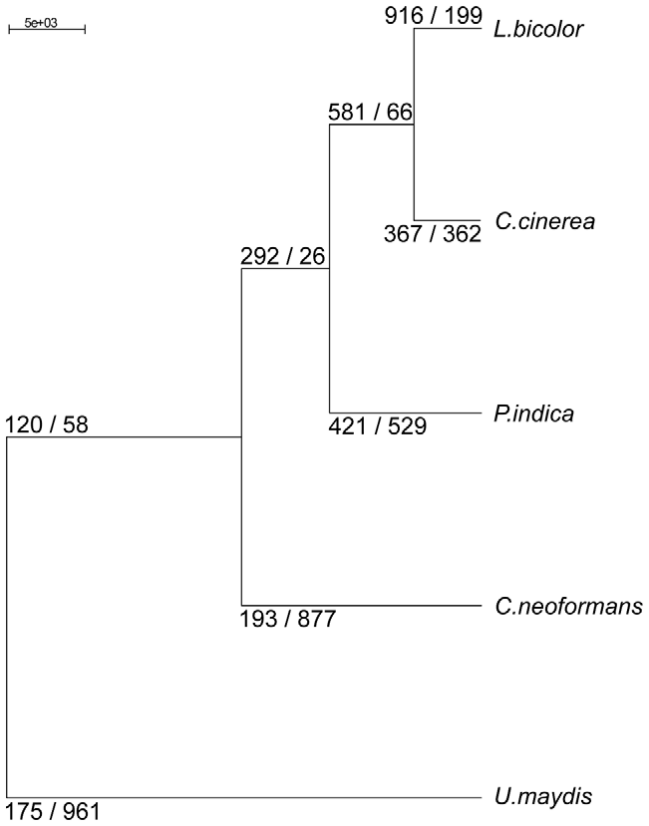
Neighbor joining (NJ) phylogenetic tree of 98 concatenated single copy genes (50,402 characters), constructed with PAUP [**104**]. Genes were selected from 245 eukaryotic core genes identified in *Piriformospora indica*, *Laccaria bicolor*, *Coprinopsis cinerea*, *Cryptococcus neoformans* and *Ustilago maydis* by blastp comparisons (eVal: 10^-3^) against the FUNYBASE [92]. The bootstrap confidence level was 100 at each internal node. Numbers indicate the amount of gene families found to be expanded/contracted by CAFE analysis [42] at the specific node as described in material and methods.

Additional analyses included clustering of protein families using Tribe-MCL [41] and the estimation of evolutionary changes in the size of these families using CAFE [42] (Table S8 and Figure 4). A total number of 4,458 multigene families were identified in the *P. indica* genome by Tribe-MCL analysis with an average of 2.26 proteins per family, which is in the expected range for the Basidiomycota (with average size of 4,488 and 2.2, respectively) and which correlated with the genome size (Table S2 and Figure S9; [30]). From the CAFE analysis, 421 families proved to be expanded in *P. indica*, 2,711 showed no change, and 529 had undergone contraction (Figure 4). In general, the domains identified by the Pfam analysis as being significantly expanded were found to be predominant in the expanded protein families, showing an overall good congruence of both methods (Table S6 and S8).

Gene families that had undergone contraction account for proteins coding for amino acid and ABC transporters (e.g. nitrate transporters, amino acid permeases, transmembrane amino acid transporter proteins, nucleobase cation symporters, ABC-2 type transporters, and CDR ABC transporters) (Table S6) and proteins involved in primary and secondary metabolism such as those for nitrate and nitrite reductase, polyketide synthase and non-ribosomal peptide synthetase (PKS, NRPS) (Table S9). Based on this data *P. indica* is predicted to experience nitrogen deficiency during growth on nitrate as sole N source. In order to test this hypothesis *P. indica* was grown on buffered minimal medium either containing no nitrogen or supplemented with N in the form of nitrate, ammonium or glutamine (Figure S10). As anticipated *P. indica* growth on nitrate is comparable to its growth on medium without N source. How the nitrogen sources impact the interaction of *P. indica* with the host is unknown and needs to be analyzed in the future.

### Carbohydrate binding domains expansion in *P. indica*

**Figure 5:**
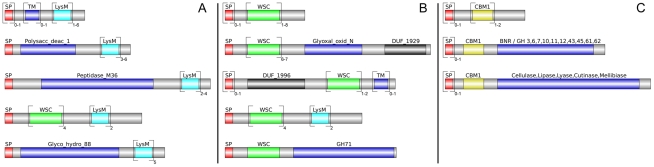
Different architecture of *P. indica* LysM and WSC containing proteins. Protein domains in the genome of *P. indica* were identified using the PfamScan perl-script (ftp://ftp.sanger.ac.uk/pub/databases/Pfam/Tools/PfamScan.tar.gz; [38]). Results were validated with SMART [101]. Domains were grouped based on their structure and visualized using DOG (version 1.0; [102]). Frequency of each domain is specified by the numbers below the brackets. A) Lectin-like LysM: 11 ORFs; LysM + polysaccharide deacetylase: 3 ORFs; LysM + peptidase M36 (fungalysin metallopeptidase): 2 ORFs; LysM + WSC: 1 ORF; LysM + glycoside hydrolase 88 (d-4,5 unsaturated β- glucuronyl hydrolase): 1 ORF; LysM + transmembrane domain (TM): 1 ORF. B) Lectin-like WSC: 28 ORFs; WSC + glyoxal oxidase: 3 ORFs; WSC + DUF 1996: 3 ORFs; WSC + LysM: 1 ORF; WSC + glycoside hydrolase 71 (α-1,3-glucanase): 1 ORF. C) Lectin-like CBM1: 14 ORFs; CBM1 + glycoside hydrolases: 32 ORFs; CBM1 + other catalytic enzymes: 15 ORFs.

**Figure 6:**
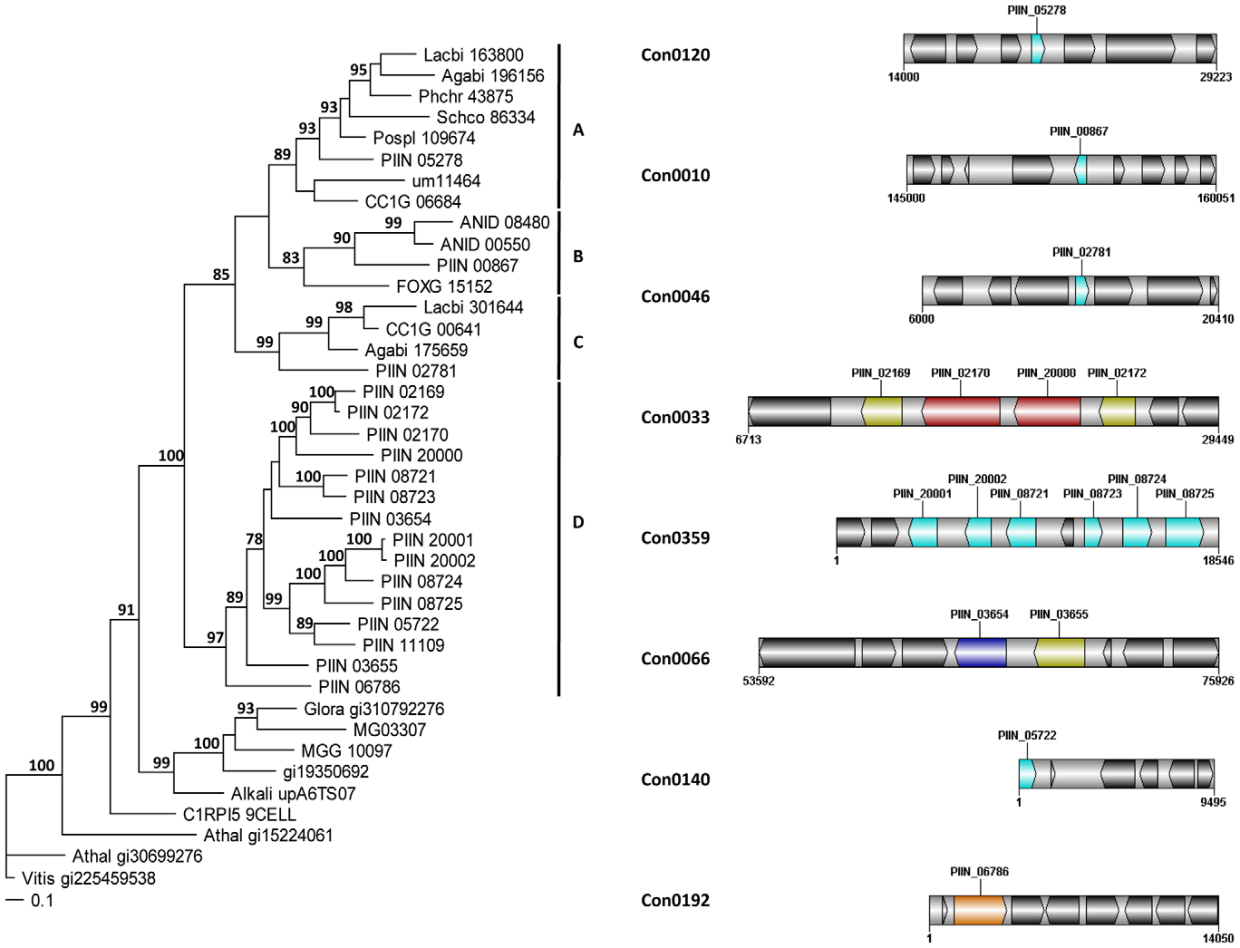
*P. indica*-specific expansion of genes encoding LysM domain containing proteins. Phylogram showing the relationships of concatenated LysM domains (left) and physical clusters of *P. indica* LysM domain containing proteins (right). MrBayes [111] analyses were conducted with the fixed (Wag) aamodel and a sample frequency of 50 with 1,000,000 generations starting the tree randomly. Split frequency was 0.0058 and PSRF 1.00. *Arabidopsis thaliana* (Athal) and *Vitis vinifera* (Vitis) were used as out-group. Bootstrap values above 70% are shown at the nodes supported. Colors in the physical clusters represent LysM proteins with similar domain architecture. Lectin-like LysM (light blue); LysM + polysaccharide deacetylase (dark yellow); LysM + peptidase M36 (red); LysM + WSC (orange); LysM + glycoside hydrolase 88 (dark blue); neighboring proteins without a LysM domain are shown in black.

**Figure 7:**
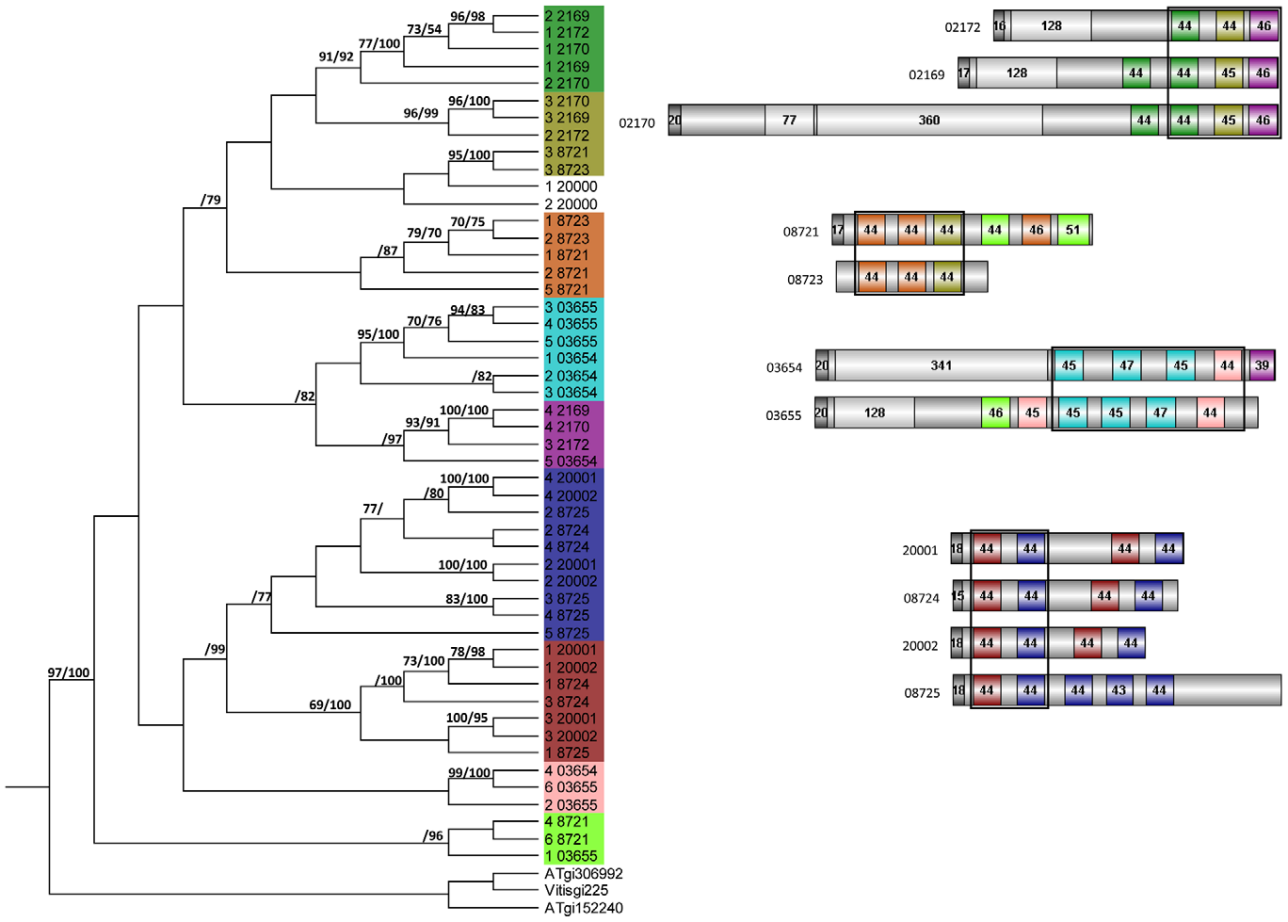
Relationship between individual *P. indica* LysM domains from 11 proteins. MrBayes and PAUP phylogenetic analyses of individual LysM domains (left); and LysM domains architecture (right) of *P. indica* proteins. MrBayes analysis was conducted with the fixed (Wag) aamodel and a sample frequency of 50 with 500,000 generations starting the tree randomly. Split frequency was 0.06 and PSRF 1.001. *Arabidopsis thaliana* (AT) and *Vitis vinifera* (Vitis) were used as out-group. NJ tree was constructed using PAUP [104] where ties (if encountered) were broken randomly, and the distance measure was the mean character difference. NJ cladogram was produced after a bootstrap analysis using maximum parsimony. MrBayes and NJ bootstrap values above 70% are shown at the nodes supported (right and left from the solidus respectively). Phylogenetically related LysM domains are shown in the same color in the tree (left) and in the schematic representation of the protein architectures (right). Signal peptides are shown in dark gray and non-LysM domains are shown in light gray. Numbers in the domains represent the number of amino acid residues of the domain as predicted by Pfamscan.

Altogether, 121 *P. indica* proteins contain either one or a combination of the following carbohydrate binding motifs: LysM, WSC or CBM1. Of these, 94 proteins are predicted to be secreted, with 21 proteins smaller than 300 aa in size. The LysM domain is a widely distributed peptidoglycan/chitin binding motif present in secreted proteins, membrane proteins, lipoproteins or proteins bound to the cell wall [43]. In bacteria the majority of the LysM containing proteins are peptidoglycan hydrolases involved in cell surface adhesion and virulence. In plants the LysM containing proteins have been found in pattern recognition receptors (PRRs) that enable the plant to identify microbial symbiotic partners or pathogens [43]. In fungi, the LysM domains are mainly associated with hydrolytic enzymes acting on fungal cell wall, but they are also present in proteins lacking other conserved domains. A lectin-like LysM protein from *Cladosporium fulvum* was found to inhibit chitin oligosaccharide triggered and PRR-mediated activation of host immunity [44]. In contrast, little information about the functions of WSC containing proteins is available [45], [46], [47]. They are thought to bind glucan and were first described in yeast as cell wall integrity sensors involved in mediating intracellular responses to environmental stress [47]. The CBM1 domain has cellulose-binding function and is almost exclusively found in fungal hydrolytic enzymes acting on plant cell walls [48]. A lectin-like CBM1 containing protein, named CBEL, was described to be involved in cell wall deposition and adhesion to cellulosic substrates in *Phytophthora parasitica* [49], [50]. The majority of *P. indica's* LysM (11 of 18), WSC (28 of 36), and some of the CBM1 (14 of 67) containing proteins are devoid of other conserved domains, resembling lectins. The rest of them are associated with different domains, which are predicted to possess plant or more rarely fungal cell wall hydrolytic activities. Figure 5 shows a schematic representation of domain combinations for the *P. indica* LysM, WSC and CBM1 containing proteins. LysM, WSC and CBM1 are short domains, containing consensus cysteine residues ([43] and Figure S11), and they are present as single or multiple repeats. Most of these proteins are predicted to be secreted, yet forms that lack a signal peptide sequence and/or have one predicted transmembrane domain were identified in the *P. indica* genome (e.g. PIIN_02781 and PIIN_07931, Figure 5). Phylogenetic analysis of concatenated LysM domains shows a strong *P. indica*-specific expansion, which include 15 of the 18 LysM proteins (Figure 6, clade D), symptomatic of a rapid evolution. Genes coding for proteins from this clade are found in clusters (of 2 to 6 genes) within the genome. The remaining 3 LysM containing proteins are distributed in 3 different clades containing Basidiomycota (A and C) and Ascomycota taxa (B). All of the LysM proteins from clade C contain one LysM domain and one transmembrane domain with no SP predicted, strongly suggesting similar functionality. Phylogenetic analysis of single LysM domains suggests that some of the domain repeats were created by sequential duplications of an ancestral domain or by the duplication of a tandem repeat (Figure 7). *P. indica* predicted ORFs containing LysM domains could be amplified by PCR from cDNA showing that all of these proteins are expressed, while pseudogenes were not found in the genome (data not shown). Furthermore transposable elements were not found in the proximity of these proteins, suggesting that unequal recombination events have contributed to gene duplication in this family. The occurrence of a protein that combines 4 WSC and 2 LysM domains (PIIN_06786) supports the hypothesis of domain reshuffling in *P. indica* (Figure 5). This domain combination is not found in closely related fungal genomes (one such protein was found in *Chaetomium globosum*, uniprot entry Q2HEN7_CHAGB) and the LysM and WSC domains present in PIIN_06786 are more closely related to other *P. indica* LysM or WSC domains respectively. These observations suggest that the most recent common ancestor of both LysM and WSC proteins most likely did not possess a protein with a combination of both domains, and the structural similarities between these proteins in bacteria, green algae, and fungi are likely due to convergent evolution.

### Assessment of *P. indica* gene expression during colonization of barley roots

**Figure 8:**
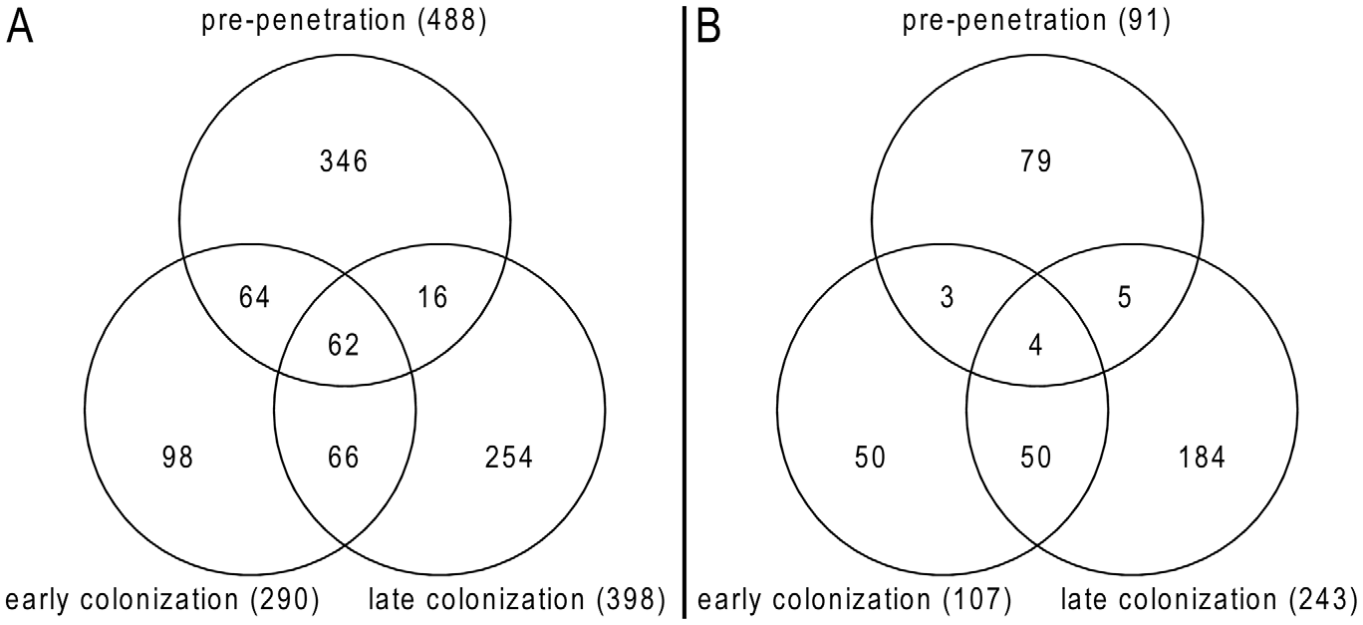
Venn diagrams showing *P. indica* genes regulated during root colonization. (A) *P. indica* genes induced during symbiosis; (B) *P. indica* genes induced during growth on dead roots at different time points of the interaction. Diagrams were created using gnuplot (version 4.4 patchlevel 2; Williams and Kelley; www.gnuplot.info).

An Agilent customized microarray was designed to monitor *P. indica* gene expression during colonization of living and autoclaved barley roots from seedlings grown on sugar-free plant minimal medium (PNM) from 36–48 hpi, 3 and 5 dpi. Fungal mycelium grown on complete medium (CM) was used as a control, because *P. indica* grew poorly on the PNM medium. Despite the fact that in young barley roots a mixture of living and dead cells were colonized by *P. indica* (Figure S1D), we found 579 genes in the pre-penetration phase (36–48 hpi), 397 genes in the early colonization phase (3 dpi), and 641 genes at 5 dpi that were differentially regulated compared to autoclaved roots (Figure 8; Table 2 and S10). These differences in gene expression are consistent with a diversified colonization strategy for living and dead roots (supported by enrichment analysis, Table S11). An interesting observation based on results from blastx searches against the NCBI nr-database (eVal: 10^−3^) emphasizes the existence of transcriptionally defined gene sets for biotrophic and saprotrophic lifestyles. Genes induced during symbiosis exhibited higher amino acid sequence similarity to those of *L. bicolor* (18% of the total induced genes). In contrast, genes induced during colonization of autoclaved roots exhibited higher amino acid similarity to those of *C. cinerea* (23%). Additionally, most of the symbiosis induced genes (40%) were non orthologous to either species but specific to *P. indica* (Table 3).

**Table 2:** Summary of induced genes during barley root colonization.

Living vs Dead	total number of induced genes	induced secreted proteins	Induced small secreted proteins
pre-penetration^1^	488	57	40 (8.2%)
early colonization^2^	290	30	23 (7.9%)
late colonization^3^	398	40	27 (6.8%)
**Living vs CM**			
pre-penetration^1^	800	127	82 (10.3%)
early colonization^2^	546	90	51 (9.3%)
late colonization^3^	567	77	40 (7.1%)

1 36 to 48 hours post infection;

2 72 hours post infection;

3 120 hours post infection.

**Table 3:** Best blast hits for the induced genes during symbiosis or saprotrophism.

Best blast hit	n° of induced genes in living roots	n° of induced genes in dead roots
*L. bicolor*	163 (18%)	68 (18%)
*C. cinerea*	119 (13%)	85 (23%)
No known ortholog	365 (40%)	101 (27%)
Others	259 (29%)	121 (32%)
Total*	906	375

*Number of genes induced at least at one time point.

**Figure 9:**
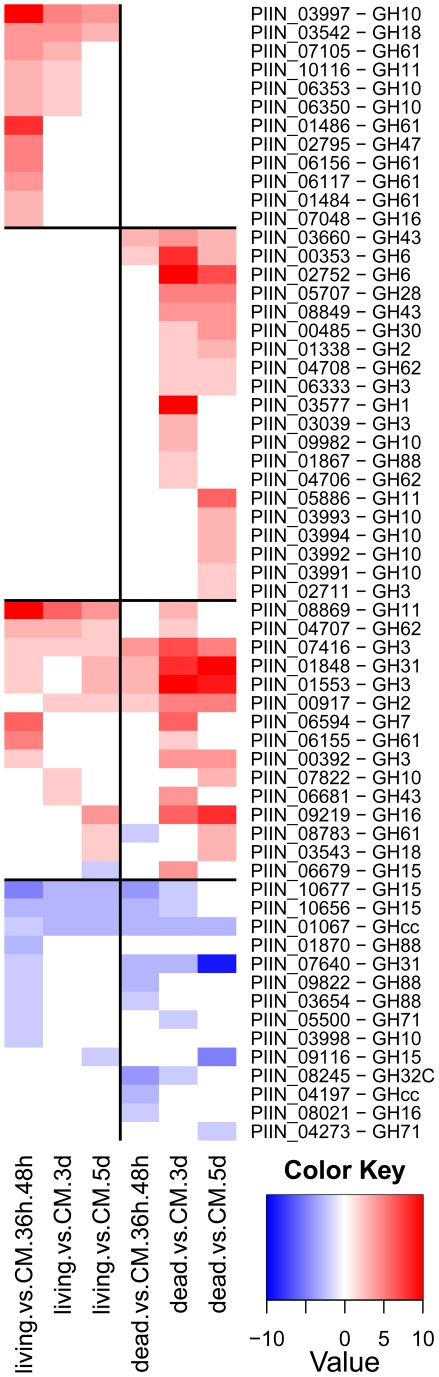
Differentially regulated *P. indica* hydrolytic enzymes. Shown are expression data from 61 (38%) of the total 160 identified hydrolytic enzymes that are at least at one time point differentially regulated in the performed microarray experiments. Heatmaps were produced using R (www.R-project.org) and are based on significant expression fold changes calculated versus complete medium (CM) control. Gene sets were manually sorted into predominantly induced in living roots; predominantly induced in dead roots; induced in both, living and dead roots to similar extent; and down regulated compared to CM.

**Figure 10:**
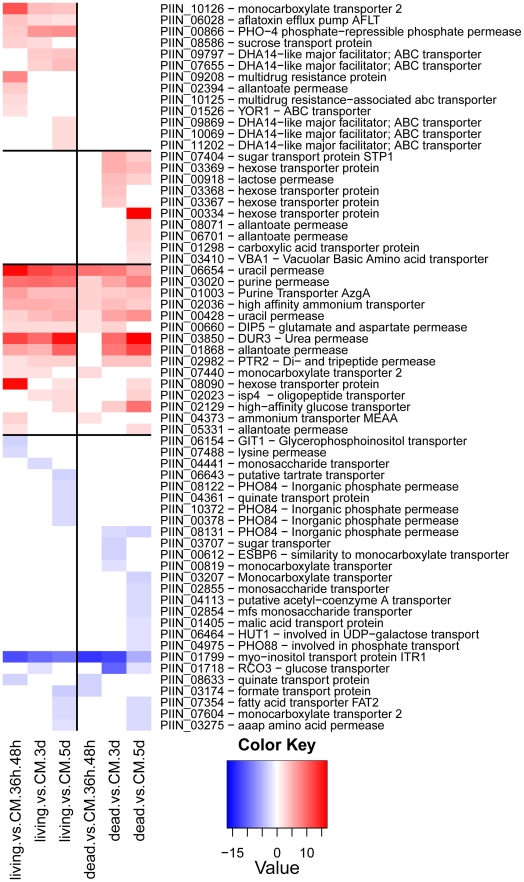
Plant responsive transporters in the genome of *P. indica*. Shown are expression data from 64 (41%) of the 262 identified transporters that are at least at one time point differentially regulated. Identification of transporters was performed manually and a comparison against the transporter classification database (TCDB) (http://www.tcdb.org/) was done. Heatmaps were produced using R (www.R-project.org) and are based on significant expression fold changes calculated versus complete medium (CM). Gene sets were manually sorted into predominantly induced in living roots; predominantly induced in dead roots; induced in both, living and dead roots to similar extent; and down regulated compared to CM.

Genes predicted to be involved in plant cell wall degradation were highly expressed at 3 dpi and remained induced or showed an even higher induction at 5 dpi on autoclaved roots (Figure 9). The high number of up-regulated genes encoding hydrolytic enzymes (including a pectin lyase, PIIN_04321, a pectin esterase, PIIN_04734 and a pectate lyase, PIIN_00890) during saprotrophic growth is consistent with the observation that colonized autoclaved roots were macerated at later stages (in contrast to non-colonized dead material). This suggests that dead tissue is subjected to intense hydrolytic activity which is not observed in colonized living roots. Nineteen genes encoding putative hexose transporters are annotated in the *P. indica* genome. Many of these genes were induced during colonization of dead roots, including a physical cluster of 3 hexose transporters with closest homology to *C. cinerea* (PIIN_03367, PIIN_03368, PIIN_03369; Figure 10). The up-regulation of genes related to carbohydrate transport and metabolism together with the induction of plant cell wall degrading enzymes (Table S10) indicate that a state of glucose depletion exists during growth of *P. indica* on dead root tissue at 5 dpi. Consistent with the existence of this state is the observation that genes for lipid metabolism were induced at this later time, while those for mitochondrial activity and biogenesis were repressed (Table S11).

**Figure 11:**
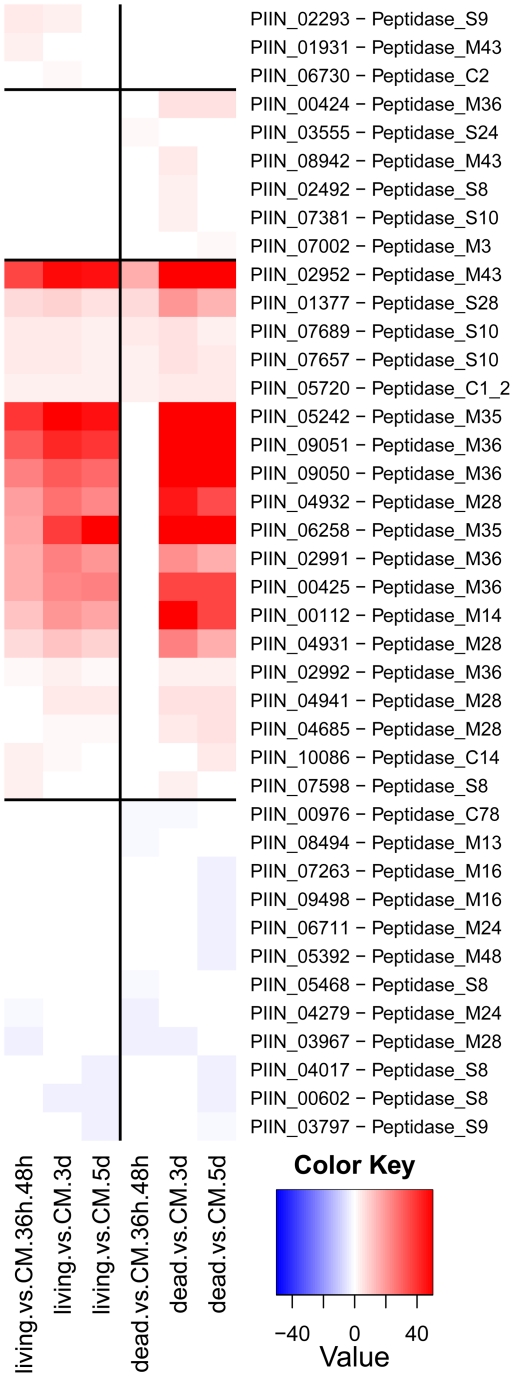
Differentially regulated *P. indica* peptidases. Shown are expression data from 40 (28%) of the total 144 identified peptidases that are at least at one time point differentially regulated in the performed microarray experiments. Heatmaps were produced using R (www.R-project.org) and are based on significant expression fold changes calculated versus complete medium (CM). Gene sets were manually sorted into predominantly induced in living roots; predominantly induced in dead roots; induced in both, living and dead roots to similar extent; and down regulated compared to CM.

Enzymes predicted to be involved in proteolysis are well represented in the *P. indica* draft genome. In particular two families of metallopeptidases, M36 (fungalysin) and M43 (cytophagalysin), are present in expanded forms. Members of these two families, together with members of the M28 (aminopeptidase Y) and M35 (deuterolysin) families, were greatly induced upon colonization of dead roots (Figure 11). The presence of a great number of metalloproteases that closely match the M36 peptidase family in *C. cinerea* ([51] and Table S6) suggests that these enzymes are involved in plant tissue degradation for nitrogen assimilation. Fungal transporter genes, involved in the uptake of different nitrogen forms, such as a urea permease (DUR3), uracil permeases, purine permeases, a high-affinity ammonium transporter, and amino acid transporters displayed a similar expression profile with increased induction over time (Figure 10). Stress response to C and N depletion may therefore be responsible for the high number of hydrolytic enzymes (CWDE and peptidases) induced at 5 dpi on dead root material.

During colonization of living roots, genes predicted to be involved in plant cell wall degradation were induced at the pre-penetration stage with a reduction in number and expression intensity at 3 and 5 dpi (Figure 9). These results suggest a tightly controlled expression of a defined set of symbiosis-related CWDE at the onset of the biotrophic phase. Production of cell wall degrading enzymes (CWDE) by plant colonizing fungi is often inhibited by glucose or other simple sugars in a well studied metabolic process known as catabolite or glucose repression [52], [53], [54]. The opposite trends observed in the expression profiles of the hydrolytic enzymes in living and in dead roots could, therefore, be partially explained by plant carbon allocation during symbiosis. Members of the expanded glycoside hydrolase GH61 enzyme family were almost solely responsive to living roots at the pre-penetration stage. Expression of GH10, GH11, GH18, and GH62 was induced at all 3 time points and may be involved in the local secretion of enzymes at the penetration site in living roots. Differences in expression of genes coding for CWDE between living and dead roots may also be explained as response to papillae formation (Figure 3 and S1A).

Expression of genes involved in protein degradation and nitrogen transport showed an increased induction over time. The expression profile for these genes resembled that observed during colonization of dead roots, although lower gene inductions were recorded for the peptidases in response to colonization of living roots (Figure 10, 11, S12 and S13). The increasing number of non-vital plant cells over time in living roots could account for this similarity in expression profile between living and autoclaved root substrate. In general, expression levels of various key genes affected by starvation, such as those involved in autophagy or coding for metacaspases, acetyl-CoA synthetase and enoyl-CoA hydratase [55], [56], were unaffected or even down regulated during symbiosis (Table S10) consistent with nutrient availability during early biotrophic interaction.

**Figure 12:**
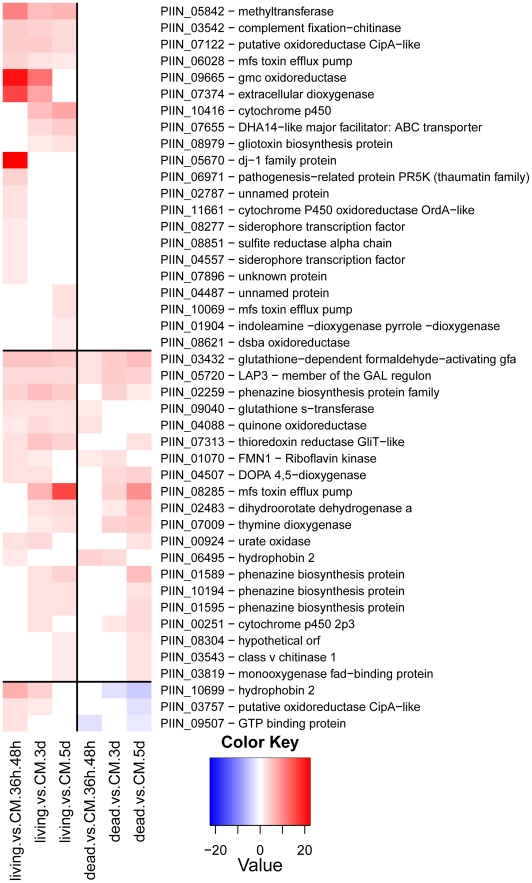
*P. indica* genes involved in stress response and secondary metabolism. Shown is a selection of 44 manually identified genes. Heatmaps were produced using R (www.R-project.org) and are based on significant expression fold changes calculated versus complete medium (CM). Gene sets were manually sorted into predominantly induced in living roots; induced in both, living and dead roots to similar extent; and down regulated compared to CM.

Fungal genes annotated in the functional categories of cell rescue and stress response were prevalent among those induced in living roots (Figure 12). An increased expression of genes involved in oxidative stress, flavonoid and phenolic compounds reduction (including a dj-1 family protein putatively identified as a catalaseA-like) and an extracellular dioxygenase was observed at the pre-penetration phase. Genes for siderophore transcription factors and a thaumatin-like protein were also up-regulated. In contrast, at later time points the fungus appears to be engaged in chemical detoxification, which involved the increased expression of genes for DHA14 and other ABC transporters, cytochrome P 450, glutathione S-transferase, isoflavone-, thioredoxin- and quinine-reductases. In addition genes with strong amino acid sequence similarity to the gliotoxin biosynthetic gene cluster of *Aspergillus fumigatus* were identified as responsive to the living substrate (e.g. gliotoxin biosynthesis protein, gliK, PIIN_08979 and thioredoxin reductase, gliT, PIIN_07313; Figure 12 [57]). Closer inspection of the microarray data showed that four additional genes (PIIN_10069 related to aflatoxin efflux pump, PIIN_10416 related to cytochrome P450, PIIN_05842 related to methyltransferase, and PIIN_08304 related to cytochrome P450) with amino acid sequence similarity to gliA, gliF, gliN, and gliC from the *Aspergillus* gene cluster were induced in colonized living roots (Figure 12). These genes were not clustered in the *P. indica* genome and the absence of a NRPS related to gliP, the key enzyme for gliotoxin production in *A. fumigatus* [57] (Table S9), suggest that the respective *P. indica* genes are involved in protection against host antibiotic compounds rather than in production of mycotoxins.

### Small secreted proteins and the identification of a novel effector family “DELD”

**Figure 13:**
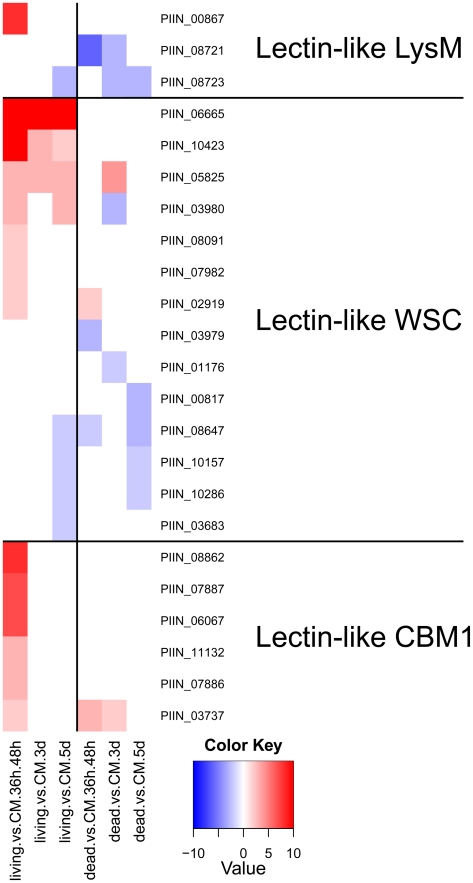
Differentially regulated *P. indica* lectin-like proteins. Shown are expression data from 23 (19%) of the total 121 identified carbohydrate binding proteins. All 23 proteins are putatively secreted and are devoid of other conserved domains, resembling lectins. Heatmaps were produced using R (www.R-project.org) and are based on significant expression fold changes calculated versus complete medium (CM). Gene sets were manually sorted based on fold changes (high to low) in living plant material at the pre-penetration stage and separated into 3 groups of lectin-like proteins: LysM (predicted to bind to chitin); WSC (predicted to bind to glucan) and CBM1 (predicted to bind to cellulose).

**Figure 14:**
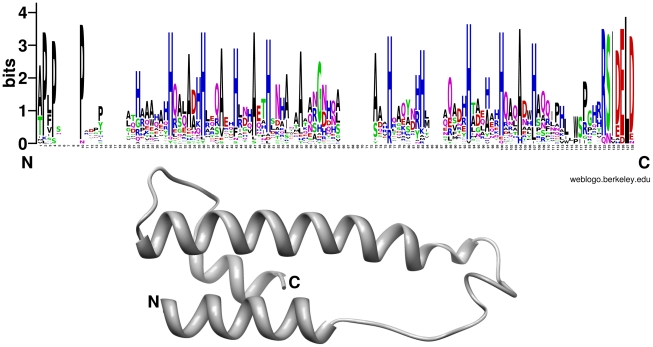
Conserved residues positions and schematic representation of the *P. indica* DELD protein structure. Regions with regularly distributed histidine (blue) and alanine (black) residues are visible in the consensus alignment (upper panel). The DELD proteins are predicted to produce two helices that are interrupted by a less conserved central region but with a conserved glycine (at position 65 in the consensus alignment). The sequence logo was created using WebLogo (version 2.8.2; [84]) based on a multiple sequence alignment of 29 DELD proteins without the predicted signal peptides using MUSCLE [112]. Secondary protein structure prediction was performed using Phyre (Protein Homology/analogY Recognition Engine) (http://www.sbg.bio.ic.ac.uk/~phyre/ version 2.0; [113]) and is exemplary shown for PIIN_05872.

**Figure 15:**
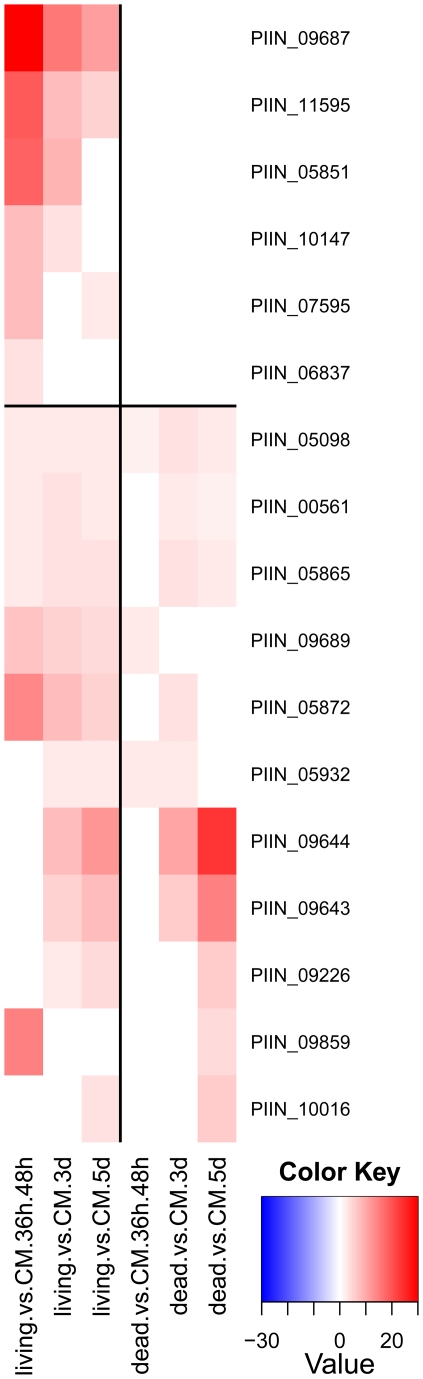
*P. indica* DELD proteins induced during colonization of barley roots (shown are 17 from the 29 identified DELD proteins). Heatmaps were produced using R (www.R-project.org) and are based on significant expression fold changes calculated versus complete medium (CM). Gene sets were manually sorted into predominantly induced in living roots; and induced in both, living and dead roots to similar extent.

It is accepted that most phytopathogenic fungi are able to reprogram plant defense and cell metabolism through the secretion of small proteins called effectors (for review see [58], [59], [60]). Recently it has been shown that effector-like proteins exist also in mutualistic fungi [61], [62]. About 10% of the genes induced during *P. indica* colonization of living barley roots encoded putative small secreted proteins (SSP, <300 aa; Table 2). Increased expression of these SSPs suggests that they are likely to play a role in determining the success of endophytic interactions that involve penetration, suppression of plant immunity and growth within living cells. Intriguingly, some of the lectin-like proteins identified in *P. indica* genome were represented within this group (Figure 13). Yet, the role played by these lectin-like proteins during symbiosis remains unclear. Since these proteins are expressed at a higher level in living roots at the pre-penetration stage, we can speculate that they are involved in modulating recognition in host-microbe interaction. This could be achieved either through mediation of adherence to host cells or, alternatively, by masking of microbe-associated molecular patterns (MAMPs) and thus avoiding recognition by the host plant. Beside these proteins, other *P. indica*-specific plant responsive SSPs with no known domains were found. A search for motifs (Table S12 and S13) in the amino acid sequences of these heterogeneous proteins identified a group of 25 proteins with a highly conserved pattern of seven amino acids “RSIDELD” at the C-terminus (named DELD) (Figure 14). Extension of this search to the genome draft recognized 4 truncated ORFs. Three of these putative genes were predicted to be pseudogenes (PIIN_10706; PIIN_10879 and PIIN_10960) and they had a higher mutation rate compared to the other DELD-encoded genes. We therefore assume that most, if not all of the DELD proteins are secreted. In total, 17 proteins containing a RSIDELD motif showed increased expression during symbiosis (Figure 15 and S12). All DELD proteins have a similar size ranging between 101 and 135 aa with no known functional protein domain. A multiple protein sequence alignment identified a conserved and regular distribution of histidine and alanine residues within the DELD proteins (Figure 14). Searches of public fungal genome databases revealed that the RSIDELD motif is present at the C-terminus in other fungal proteins but, when present, the proteins bearing this motif are not highly enriched in histidine and alanine residues (Table S12). Interestingly, two ectomycorrhiza-regulated small secreted proteins from *L. bicolor* possess a DELD motif at the C-terminus but lack a high content of histidines. This observation supports the notion that the central part of the protein and the C-terminal tail are functionally distinct entities. Secondary structure prediction shows that the DELD proteins most probably form a two-helix bundle interrupted by a central conserved glycine residue (Figure 14). Amino acid sequence similarity searches with the central part of the DELD proteins revealed a ∼30% sequence identity with HRPII, a protein family from *Plasmodium falciparum.* This similarity was primarily due to the high histidine and alanine content (Figure S14). HRPII is an abundant protein released during erythrocyte infection by the malaria parasite and was reported to be localized in several cell compartments including the cell membrane and the cytoplasm of the host cells [63]. HRPII has been implicated in the detoxification of heme [64], in cytoskeleton modification by actin binding [65] and in inhibition of antithrombin (AT) by selectively binding to coagulation-active glycosaminoglycans (such as dermatan sulfate, heparin sulfate and heparin) in a Zn^2+^ dependent manner [66]. Further this protein was shown to be able to bind to phosphatidylinositol 4,5-bisphosphate (PIP_2_) and erythrocyte ghosts by undergoing a coil-to-helix transition [65]. Although the function of the HRPII seems to be still controversially discussed, this is one of the best studied histidine rich protein at the present time. The function of histidine and alanine rich proteins in fungi is not known.

We investigated the association between the DELD gene family and transposable elements by assessing the extent to which they occurred together in the *P. indica* draft genome sequence. In contrast to the LysM protein-coding genes, the occurrence of DELD sequences strongly correlates with the presence of flanking transposable elements in gene-poor genomic regions (Figure S15). Similar to effectors found in other filamentous organisms [67], [68], [69], the expansion of DELD genes in *P. indica* may be accounted for by transposition activity. These findings suggest that the DELDs represent a new gene family with a conserved domain of unknown function secreted during symbiotic root colonization.

### Conclusions

*P. indica* possesses a small genome that is gene dense with few repetitive DNA sequences. Despite the unusual low number of transposable elements in the *P. indica* genome compared to known plant pathogens and symbionts [28], [67], [30], a high number of expanded gene families exist, which are typically present in clusters (of 2 to 7 genes) within the genome. Expansion of these families is likely to be due to local duplication events caused by unequal recombination, rather than retrotransposition. An exception to this is the expansion of the *P. indica*-specific DELD protein-coding gene family. All members of this novel family occurred in the proximity of transposable elements strongly suggesting a significant co-expansion between DELD paralogs and transposon sequences that benefited *P. indica* in some way during adaptation to the endophytic growth. This gene family expansion together with the combined rapid evolution of different types of plant responsive lectin-like proteins and different classes of secreted CWDE must have provided important functional advantages in the colonization of different plant hosts, e.g. by overcoming host inhibitors and by minimizing MAMP-triggered immunity (MTI) induction. Consistent with this hypothesis, recent work has shown that *P. indica* has evolved an extraordinary capacity for plant root colonization that has been attributed to its potential to suppress host MTI [19]. Future research is required to elucidate the contribution of these protein families to *P. indica's* colonization strategy.

The facts that *P. indica* can grow readily on synthetic media and can colonize a wide range of mono- and dicotyledonous plants, indicate that its genome did not undergo host driven specialization as observed in typical obligate biotrophs [28]. Further, the observed dual ability of *P. indica* to colonize living and dead cortex cells point to a widening of the symbiotic lifestyle, i.e. implementing, maintaining or enforcing properties of biotrophy and saprotrophism, which maybe a reason leading to a broader host range. In agreement with this hypothesis, extended comparative analysis of *P. indica* genomic and transcriptomic traits with those of other Ascomycota and Basidiomycota taxa with different lifestyles decoded features typically associated with biotrophism [26], [28]. These were the presence of small secreted proteins during symbiosis and the absence of genes encoding for nitrate uptake and reduction, as well as those for secondary metabolism, such as polyketide synthase and non-ribosomal peptide synthetase. On the other side, the genome sequence uncovered saprotrophic features uncommon to symbionts, i.e. expansions in cell wall degrading enzymes and metallopeptidases [70]. The tightly controlled expression of CWDE and the identification of different lifestyle-associated genomic traits argue for a biphasic lifestyle. This interpretation of the genomic information is supported by microscopic data that revealed an early biotrophic growth followed by a cell death-associated phase. In contrast to hemibiotrophic pathogens, such as *Magnaporthe oryzae*, where the switch from an initial biotrophic growth to necrotrophy leads to disease symptoms [31], [55], the interaction of *P. indica* with plant roots has a beneficial outcome for its host. It remains to be clarified whether the beneficial effects produced by *P. indica* on its host are merely attributable to the biotrophic phase or to a yet unknown mechanism associated with the lifestyle switch.

The finding of a mutualistic symbiont with a biphasic lifestyle support the idea that the evolution of diverse mycorrhizal associations present in the order Sebacinales have begun with saprotrophic fungi that became endophytic, and then progressed to obligate biotrophic forms. Genome sequencing of other sebacinoid species is ongoing and will help clarifying, at least for this group of fungi, the evolutionary steps involved in mycorrhizal symbiosis. The availability of the genome and the genetic tractability of *P. indica* will provide powerful experimental advantages for investigating fundamental aspects of symbiosis, including functional analyses of the effector-like proteins and symbiosis determinants, identification of novel symbiosis/pathogenicity genes by genome comparison, population genomics, and SNP polymorphism of symbiosis-regulated genes.

## Materials and Methods

### RNA-Seq, genome sequencing and assembly

Total RNA was extracted with TRIzol reagent (Invitrogen, Darmstadt, Germany) from germinating *Piriformospora indica* (DSM 11827, DSMZ, Braunschweig, Germany) chlamydospores (24h) and from 3 days old mycelium grown in liquid complete medium (CM) [36] and pooled together. Messenger RNA (mRNA) containing poly-A tails were isolated from 500 µg of this pool using MN-Nucleotrap mRNA Kit (Macherey-Nagel, Düren, Germany). After a precipitation step with isopropanol and dilution in milliQ water, first strand cDNA was prepared using a SMART RACE cDNA amplification Kit (Clontech/Takara Bio Europe, Saint-Germain-en-Laye, France) according to the manufacturer's protocol. SMART oligo II and 3′ RACE CDS primers (Clontech) were used for first strand cDNA synthesis. The cDNA reaction mixture was precipitated with isopropanol and dissolved in milliQ water to a final concentration of 100 ng/µl. A 1.5 µl aliquot was used for first strand cDNA normalization using the Evrogen JSC Kamchatka crab duplex specific nuclease, DSN (BioCat GmbH, Heidelberg, Germany) as described before [71]. After DSN inactivation long distance PCR with primers compatible to the adapters using a proofreading taq polymerase was performed as follows: 95°C for 1, min, twenty-seven PCR cycles at 95°C for 15 s, 65°C for 30 s, 72°C for 3 min and one cycle at 72°C for 7 min. Finally 40 µl of the solution (255 ng/µl) were sent to Roche Diagnostics Corporation (454 Life Sciences) for pyrosequencing using the 454 platform.

Genomic DNA was extracted from 10 g fungal material grown in CM liquid culture using the CTAB protocol of Doyle and Doyle [72]. Sequencing of the genome of *P. indica* was performed by Eurofins MWG operon, Ebersberg, Germany, using the 454 GS FLX Titanium platform. The performed paired-end pyrosequencing resulted in 1.406.954 reads with 45.392 mate pair candidates. Assembling of the data was accomplished by using the Celera Assembler (version 5.3, [73]) and the CABOG pipeline [74] to reduce assembly problems caused by long homopolymeric stretches in the reads. An additional assembly of these contigs was performed by making use of the mate pair information. The final genome set consists of 1.884 scaffolds. 7945 degenerate contigs were excluded from the assembly because they failed different quality criteria, e.g. they had low sequence support (high proportion of bases with low PHRED value [75]) or a length below 1 kb.

### Raw sequence analysis

GC-content, length and average coverage of both scaffolds and contigs were analyzed by plotting GC-content and average coverage against the contig length using gnuplot (version 4.4 patchlevel 2, Williams and Kelley, Figure S7). Most of the contigs share a coverage of about 22 fold (21.74 for contigs) and a GC-content of about 50% (49.78% for contigs, 50.68% for scaffolds). Additionally, 15 contigs had a high coverage of 200 fold and a low GC-content of about 26%. These contigs could be assembled into the circular mitochondrion of *P. indica* (see material and methods, analysis of the mitochondrion). Contigs with a lower coverage of about 10 fold were also identified. The high number of scaffolds despite use of deep sequencing and the differences in the coverage of the contigs resemble the assembly challenges and coverage differences in the genome project of the diploid human pathogen *Candida albicans* [37] giving a first indication for the presence of two genomes in *P. indica.*

### Identification of single nucleotide polymorphisms

The presence of two haploid genomes was bioinformatically verified by searching for single nucleotide polymorphisms (SNPs) using the swap454 program from the Broad Institute [76]. According to the protocol (http://www.broadinstitute.org/science/programs/genome-biology/computational-rd/454-help) a new standard flowgram format (SFF) file was created from the raw read sequence fasta and quality files. For the creation of a coverage map the Celera-assembled contig sequences were used as reference. The SNP calling parameters were chosen in such a way that at least 10% of the reads had to differ from the reference sequence in order to be counted as a SNP. With this procedure a total of 61.532 SNPs could be identified in the genome (Table S5) of which 1.039 (1.7%) were identified on degenerate contigs and therefore discarded from further analysis. For the validation of the prediction, the number of SNPs per contig was plotted against its size using gnuplot 4.4.2. (Figure S8). The plot shows a proportional relation between the number of SNPs in a contig to the size of the contigs (R^2^ = 0.8625) which is a first hint of a good reliability of the prediction. Additionally, the predicted number and position of SNPs in the contigs was manually validated in ∼100 randomly chosen contigs using the assembly viewer eagleview [77] with a high degree of consistency (∼95%). Further, the SNP prediction from the contigs was mapped onto the scaffolds. By doing so, few problems were encountered. First, small contigs without SNPs were occasionally assembled together with contigs with SNPs resulting in a mixture of both datasets. Second, the scaffolds contain a significantly higher number of unknown nucleotides (“N's”) than contigs (212090 vs 270) because of the performed mate pair assembly. These nucleotides could not be considered in the SNP calling. These data are therefore not shown.

From all genes that were predicted from the *P. indica* genome, 1056 (8.97%) were found in contigs that did not contain any SNPs. 110 of these genes (10.42%) had a signifcant hit against the NCBI nr-database (eVal: 10^−3^).

### *P. indica* Transposable Elements (TEs)

RepeatScout [78] was used to identify *de novo* repetitive DNA in the *P. indica* genome draft. The default parameters (with l = 15) were used. RepeatScout generated a library of 913 consensus sequences. This library was then filtered as follows: 1) all the sequences less than 100 bp in size were discarded; 2) repeats having less than 5 copies in the genome were removed (as they may correspond to protein-coding gene families) and 3) repeats having significant hits to known proteins in Uniprot [79] other than proteins known as belonging to TEs were removed. The 227 consensus sequences remaining were annotated manually by a tblastx search [80] against RepBase (http://www.girinst.org/repbase/index.html). Five sequences have homologies with Class 1 retrotransposons LINE and three with Class 1 LTR retrotransposons copia. Since Class 1 retrotransposons gypsy was not identified in the RepeatScout repeat library and such elements are largely represented in fungi, a rpsblast search [80] with the reverse transcriptase 1 (RVT1) motif (pfam00078) found in Class 1 retrotransposons gypsy was preformed. The 21 putative RVT1 sequences obtained with the rpsblast search were compared by a tblastn search against RepBase. Sixteen sequences have homologies with Class 1 retrotransposons LINE, three with Class 1 retrotransposons Gypsy, one with Class 1 retrotransposons copia and one did not have homology. To identify full length LTR retrotransposons, a second *de novo* search was performed with LTR_STRUC [81]. No full length LTR retrotransposons were identified. The number of TE occurrences and the percent of genome coverage were assessed by masking the *P. indica* genome assembly using RepeatMasker [82] (www.repeatmasker.org) with the 227 consensus sequences coming from the RepeatScout pipeline. RepeatMasker masked 4.68% of the *P. indica* genome assembly. 4.12% of the genome was masked by repeated elements belonging to unknown/uncategorized families (Table S3).

### *P. indica* SSR

MISA (http://pgrc.ipk-gatersleben.de/misa/download/misa.pl) was used to identify mono- to hexanucleotide Simple Sequence Repeat (SSR) motifs using default parameters. A total of 602 SSRs have been identified in the *P. indica* genome corresponding to 213 mono-, 154 di-, 218 tri-, 4 tetra-, 2 penta- and 11 hexanucleotide motifs. The relative abundance of SSRs was calculated as the number of SSRs per Mb. For all 602 SSRs, the relative abundance was 24 SSRs/Mb.

### Transfer RNAs / codon usage

For the prediction of tRNAs the program tRNAscan-SE (version 1.23, [83]) was used. The prediction was performed on the nucleotide sequences of scaffolds and contigs with the default search mode and eukaryotic gene model. In total 52 standard proteinogenic tRNAs could be identified from which 37 contained introns. Additionally, 2 tRNAs of an unknown isotype and 4 pseudo-tRNAs were predicted by tRNAscan (Table S4).

Codon triplets and a corresponding codon table of *A. bisporus, A. nidulans, C. cinerea, C. neoformans, F. oxysporum, H. annosum, L. bicolor, M. larici populina, P. crysoporium, P. indica, P. ostreatus, P. placenta, P. graminis, S. commune, S. lacrymans, S. roseus, T. atroviride, T. mesenterica, T. reesei* and *U. maydis* were calculated from nucleotide sequences of the predicted genes using the programming language JAVA (http://www.java.com/en/). The codon triplets were then used to calculate frequency plots using WebLogo [84]. The plots show which nucleotide is preferred in each position of the codon triplets and indicate that despite the low number of tRNAs *P. indica* has very similar codon-usage preferences to those of *C. cinerea*, *P. ostreatus, T. atroviride* and *A. nidulans*. (Figure S4). A list of all reference genomes used in this study can be found in Table S14.

### Gene modelling

Gene modelling for *P. indica* was done by applying 3 different gene prediction programs: 1) Fgenesh [85] with different matrices (trained with *Aspergillus nidulans*, *Neurospora crassa* and a mixed matrix based on different species); 2) GeneMark-ES [86] and 3) Augustus [87] with *P. indica* ESTs as hints and default gene models for *C. neoformans*, *U. maydis*, *C. cinerea* and *L. bicolor*. In addition, 857 yeast proteins from CYGD [88] were mapped to the *P. indica* contigs using Exonerate [89] to help define genes. The mapped genes were used to retrain Augustus (starting with parameters from the default *L. bicolor* model) and subsequently predict new genes. Putative genes were also considered by first mapping annotated proteins from *U. maydis*, *L. bicolor* and *C. cinerea* onto the *P. indica* genome using Exonerate and then accepting only those *P. indica* genes that could be mapped back to the original gene structure from the homologous organism. The different gene structures were displayed in GBrowse [90] allowing manual validation of all coding sequences (CDSs). Annotation was aided by blastx hits between the *P. indica* genome and those from *L. bicolor*, *C. cinerea* and *U. maydis*, respectively. The best fitting model per locus was selected manually and gene structures were adjusted by manually splitting them or redefining exon-intron boundaries based on EST data where necessary. A final set of 11769 protein coding genes were predicted from the *P. indica* genome.

### Evaluation of gene modelling

10350 ESTs were assembled from 454 generated RNA-Seq reads. ESTs were mapped onto the genome using Blat [91]. Evaluation of annotated introns was done against introns defined by ESTs. For 100% identity mapped ESTs without gaps, the sensitivity is ∼89% and specificity is ∼97%. The performance drops to 87 and 95% sensitivity and specificity, respectively for imperfectly mapped ESTs (Table S1). Furthermore, the predicted protein set was searched for highly conserved single (low) copy genes to assess the genome completeness. Ortholog genes to 245 of 246 single copy genes could be identified by blastp comparisons (eVal: 10^−3^) against the single-copy families from all 21 species available from the FUNYBASE [92]. Additionally, 245 of 248 core-genes commonly present in higher eukaryotes (CEGs) could be identified by blastp comparisons (eVal: 10^−3^) [34], [35].

### Annotation of predicted open reading frames and comparative analysis

The 11769 protein coding genes of *P. indica* were analyzed and functionally annotated using the PEDANT system [93], accessible at http://pedant.helmholtz-muenchen.de/genomes.jsp?category=fungal. The corresponding GBrowse set is located at http://mips.helmholtz-muenchen.de/gbrowse/fungi/cgi-bin/gbrowse/piindica/. The genome and annotation was submitted to the EBI (http://www.ebi.ac.uk/GOA/RGI/index.html) and can be found under the accession numbers listed in Table S17.

For comparative analysis the *P. indica* proteome and those of four related basidiomycetes, *L. bicolor*, *C. cinerea*, *U. maydis* and *C. neoformans,* were analyzed using the following tools. 1) Secreted proteins were predicted using TargetP and SignalP as described in material and methods, amino acid motifs in *P. indica*; 2) Gene ontologies (GO) were assigned using Blast2GO [94]; 3) The percentages of assigned GOs in level 4 of molecular function were calculated for the secretome of each of the four related fungi and used for comparative analysis.

### Sub cellular localization of predicted proteins

Cellular targets of the *P. indica* proteins were predicted by WoLF PSORT (version 0.2, [95]). To improve the accuracy of the program the final output was filtered by allowing predictions only if the “first neighbour” was more than 50% higher than the “second neighbour”. A putative subcellular localization could be assigned to 6.341 proteins (Table S15).

### Prediction of secreted proteins

The prediction of secreted proteins was performed by using the TargetP software package v1.1 [96] (including cleavage site predictions by SignalP, [97]) with standard settings for non-plant networks. 1.846 proteins were predicted to contain a signal peptide which targets them to the secretory pathway. This set was further refined by excluding all proteins with a low reliability class from the TargetP prediction (3–5) as well as proteins which contain more than one transmembrane domain according to TMHMM v2.0 [98] prediction with standard settings. In total 867 proteins were assigned to the secretome of *P. indica*.

### Amino acid motifs in *P. indica*

In order to screen the genome of *P. indica* for known and unknown motifs in the amino acid sequence, a self-written JAVA program based on regular expressions was used which was initially trained on the frequently described oomycetes effector motif “RXLR…EER” [99]. Including three different derivatives of this motif 321 (309 degenerated) RXLR-like motifs could be found in the genome of *P. indica.* However, only 5 proteins with a degenerated motif possess a signal peptide and none of them were found to be up-regulated during colonization of barley roots (Table S13 and S10).

Further a yet undescribed C-terminal motif with the strongly conserved consensus sequence “RSIDELD” motif could be identified in 29 proteins annotated in *P. indica*. All of these proteins are less than 135 amino acids in size and contain a significantly increased number of regular distributed alanines and histidines but no cysteines (compared to the whole proteome; p<0.01). To confirm the uniqueness of this motif to *P. indica*, a psi-blast [100] against the NCBI nr-database as well as a screening against all reference genomes was performed (Table S12).

While the blast search produced only a few hits of low reliability, the motif search identified 43 putative RSIDELD motifs in all genomes of the reference set within 20 bp of the C-terminal region. However, several of the identified motifs differ, in contrast to those from *P. indica*, significantly from the consensus and none of the proteins showed the regular histidine/alanine distribution or even the increased concentration of these amino acids compared to the DELD proteins from *P. indica* (Table S12).

### LysM and WSC proteins in *P. indica*

LysM and WSC protein domains were identified in the genome of *P. indica* and all other fungi from the reference set by using the PfamScan perl-script [38], ftp://ftp.sanger.ac.uk/pub/databases/Pfam/Tools/PfamScan.tar.gz) and the results were validated with the SMART [101] analysis pipeline using standard settings. The 18 LysM and 36 WSC proteins from *P. indica* were grouped based on their domain structure and visualized using DOG (version 1.0, [102], Table S7, Figure 5). Because the combination of LysM with other domains is unusual in *P. indica* the prediction of all 18 genes was verified by PCR on genomic DNA and cDNA.

For phylogenetic analysis the LysM and WSC domains of each protein were extracted and concatenated by a self-written JAVA program. LysM and WSC nucleotide (nt) and deduced aa sequences were aligned in 4 datasets together with publicly available sequences obtained from GenBank (http://www.ncbi.nlm.nih.gov), PFAM (http://pfam.sanger.ac.uk/) or individual genome sequencing projects. All alignments were constructed at the nt and aa level using ClustalX version 1.83 [103] and then manually corrected as needed using BioEdit (http://www.mbio.ncsu.edu/bioedit/bioedit.html). Phylogenetic analyses were performed in two steps. First all available sequences were included in neighbour joining (NJ) (nt and aa) and maximum parsimony analysis (nt) using the program PAUP [104]. The LysM alignments contained data from 186 taxa whereas the WSC alignments contained data from 126 taxa. Parsimony search consisted of 1,000 rounds of random stepwise sequence addition with all changes weighted equally and bootstrap analyses consisting of 1,000 replicates in heuristic search with random sequence addition (10 replicates). Heuristic searches were performed using random sequence addition (up to 50 replicates) and the tree-bisection reconnection (TBS) branch-swapping algorithm. A consensus of multiple trees was performed by majority role and collapsed when conflict present. NJ (nt and aa) analyses were conducted utilizing the GTR + I + G model with parameters estimated by the program and 10,000 bootstrap replicates or mean character difference. A selection of the closest related sequences was done based on the results obtained from the PAUP phylogenetic analysis of nt and aa alignments. Selected aa sequences were used in a final analyses of single and concatenated domains performed with MrBayes with the fixed (Wag) aamodel and a sample frequency of 50 with 500000 and 1000000 generations starting the tree randomly (Figure 6 and 7). The aa alignment of concatenated LysM sequences contained data from 40 taxa and a data matrix of 306 characters whereas the aa alignment of individual LysM domains contained data from 50 *P. indica* domains and 3 plant domains and a data matrix of 59 characters. The aa alignment of concatenated WSC sequences contained data from 50 taxa and a data matrix of 794 characters, whereas the aa alignment of individual WSC domains contained a selection of 44 domains and a data matrix of 93 characters.

### Cluster analysis, MCL

Clustering of proteins was performed using mcl (version 10–201, [41]) according to the online available workflow protocol (http://micans.org/mcl/man/clmprotocols.html#blast). The inflation parameter was defined by clustering with increasing inflation parameters going from 1 to 4 in steps of 0.2. All results were compared with respect to their ability to group LysM and WSC proteins seperately while clustering only *P. indica* proteins. Based on these results an optimal inflation parameter of 1.4 was used for all further clustering procedures.

To identify *P. indica* specific protein families in the basidiomycetes group, a blastp (eVal: 10^−3^) “all vs all” comparison of the proteomes of *P. indica*, *L. bicolor*, *C. cinerea*, *U. maydis* and *C. neoformans* was performed and used as input for the mcl workflow. Within this group, 6704 protein families were identified containing at least two proteins. 355 of these clusters were *P. indica* specific. The *P. indica* specific protein families containing 10 or more proteins were manually revised in terms of secretion, regulation during colonization of barley roots and amino acid composition. Almost all of these protein families consisted of moderately to strong plant responsive genes. All 29 DELD proteins occurred in cluster 144 (37 proteins in total). Additional analysis of the remaining 8 proteins in the group showed either a similar expression pattern or a similar amino acid composition in comparison to the DELD proteins but they did not possess the 7 aa conserved motif. It is still possible that these proteins have a similar function as the DELD proteins and share therefore a certain degree of similarity which groups them together.

### Cluster analysis, protein domains

Clustering of proteins was performed based on predicted functional domains. Protein domains were predicted on the proteomes of *C. cinerea, L. bicolor, U. maydis, C. neoformans, P. graminis, T. reesei, A. nidulans, F. oxysporum* and *T. melanosporum* using the PfamScan perl-script. To determine decreased/increased number of proteins in comparison to the other genomes, chi-square-statistics were applied using R (http://www.R-project.org) and the whole dataset was filtered for domains with an adjusted significance value of p<0.005 (Table S6). All clusters with a domain number below 5 were discarded. In the resulting data set *P. indica* protein domains were considered to be enriched when they had the highest number in comparison to the other genomes or to a subset of genomes grouped by lifestyle or phylum. On the contrary, *P. indica* protein domains were considered to be constraint when *P. indica* had the lowest or second lowest number of protein members in comparison to the other genomes (Table S6).

### Evolutionary analysis of protein families (CAFE)

Evolutionary changes in protein family size were analyzed using CAFE (version 2.2, [42]). For the identification of protein expansions/contractions, all protein families from the MCL analysis were used that contained at least 5 proteins. From this set, all protein families that are unique to one of the analyzed genomes were excluded. A phylogenetic tree was constructed based on 98 single copy genes from *P. indica*, *L. bicolor*, *C. cinerea*, *C. neoformans* and *U. Maydis*, predicted as described in material and methods, evaluation of gene modelling.

The CAFE analysis included 3,661 protein families (from 4,458). From these, 421 families were expanded in *P. indica*, 2,711 showed no change and 529 families had undergone contraction. Table S8 shows the 62 largest expanded protein family clusters in *P. indica*. A comparison of the CAFE results to those from the Pfam domain clustering shows the overall good agreement of both methods but reveals also the drawbacks and the necessity to use both methods. The Pfam domain clustering uses no phylogenetic information and counts proteins with different domains multiple times. The MCL/CAFE approach used phylogenetic information and protein similarities but is unable to successfully cluster all functionally related proteins into distinct families.

### Analysis of the mitochondrion

For the assembly of the *P. indica* mitochondrion, all contigs with either a high coverage or a low GC-content (Figure S7) were assembled in a single scaffold with a length of 63.682 bp and a GC-content of 26.29%, using the contig assembler seqMan [105]. Circularity was verified by PCR with primers designed at the beginning and at the end of the scaffold. Genes on the mitochondrion were predicted using a program pipeline with different bioinformatical tools. 1) Different *in silico* sheared fragments were analyzed by Blast2GO to identify all genes on the mitochondrion of *P. indica*. The exon/intron structure of these genes was then refined by building consensuses from multiple sequences alignments produced by the program protein2genome of the Exonerate package. A manual revision of the predictions resulted in a full set of proteins that are commonly present in fungal mitochondrions (Figure S16).

### Microarray experimental design

*P. indica* is able to colonize living plant roots as well as dead plant material. In order to address the fungal gene expression in these two unequal environments, experiments were performed with *P. indica* growing on living and dead barley roots. *P. indica* was cultivated on complete medium agar plates or liquid medium as described before [36]. Barley seeds (*Hordeum vulgare* L. cv. Golden Promise) were surface sterilized with 3% sodium hypochlorite, rinsed in water and pregerminated for 3 days in dark. For inoculation of barley roots with *P. indica*, the roots were dipped in a chlamydospore suspension (500,000/ml in 0.05% Tween water) or mock inoculated and grown in sterile culture on a minimal medium (1/10 PNM) and under same growth chamber conditions as described in [22]. To address the experimental design four different treatments were done (*P. indica* on barley roots on 1/10 PNM medium, *P. indica* on autoclaved barley roots on 1/10 PNM medium, *P. indica* on 1/10 PNM medium and *P. indica* on CM medium), each in three independent biological replications. Root and fungal material was harvested in liquid nitrogen after 24, 36, 48, 72, 120 and 168 hpi. For each time point roots from 15 to 20 living plants or 21 to 36 autoclaved plants were pooled. Total RNA was extracted with TRIzol (Invitrogen, Karlsruhe, Germany) following the manufacturer's instructions. RNA quality was analyzed with a 2100 Bioanalyzer (Agilent, Santa Clara, USA). Two independent biological replicates for each treatment were labelled for microarrays analysis. RNA from the time points 36 and 48 hpi of *P. indica* colonizing roots were pooled together and referred to as the pre-penetration sample. Two more time points were selected for the hybridization, 72 hpi (early colonization) and 120 hpi (late colonization). Further RNA from 36, 48, 72 and 120 hpi of *P. indica* grown on CM or PNM were pooled together and used as controls, giving a total of 16 samples. The labelling preparation was performed according to Agilent's One-Color Microarray-Based Gene Expression Analysis (Quick Amp Labeling) with Tecan HS Pro Hybridization protocol (version 6.0). For each reaction 500 ng of total RNA from each experiment was used. Cye-3-labeled probes were afterwards hybridised to 2×105k custom-designed Agilent microarrays according to Agilent's One-Color Microarray-Based Gene Expression Analysis (Quick Amp Labeling) protocol (version 5.7). The microarray design was performed using eArray (https://earray.chem.agilent.com/earray/). Up to six 60-mer probes were calculated with the best distribution methodology. Additionally, probes for 265 barley genes (including genes involved in defense and transport), 158 *A. tumefaciens* genes (bacterial control) and 11 *P. indica* housekeeping genes (positive control) were generated. To evaluate the hybridization efficiency within one array, probes from 10 *P. indica* genes were hybridised randomly in 10 replicates.

Microarray image files were analyzed using Agilent's Feature Extraction software v. 10.5. For each spot, signal and background intensities were obtained. To allow for comparison of expression levels across experiments, the raw data were standardized by quantile normalization. To assess the quality of the slides diagnostic plots were generated. Intensities from same-nucleotide probes were averaged. In each group-comparison the log2-ratio between corresponding intensities was calculated and averaged over all probes of an ORF. The Students t-statistic was applied to test ORF signal averages for significant differences between groups. Probes with low reproducibility in the two experiments were discarded from further analysis. The selection of differentially expressed genes is based on a fold change of 2 and an absolute t-statistic of 1.96. Preliminary analysis of the microarrays data indicated that *P. indica* grown on 1/10 PNM was under conditions of severe starvation, therefore the data from this control were not further used in our study. Gene annotations and expression data from *P. indica* grown on complete medium and from living and autoclaved barley roots colonized by *P. indica* are stored in Gene Expression Omnibus (http://www.ncbi.nlm.nih.gov/geo/) under the accession number GSE31266 (http://www.ncbi.nlm.nih.gov/geo/query/acc.cgi?token=hdabzwmswaqkmxs&acc=GSE31266), which complies with MIAME (minimal information about a microarray experiment) guidelines. The R environment and the Bioconductor package ‘Limma’ was used for quality control and normalization of the data.

### Verification of microarray results

Microarray data were verified by quantitative real-time PCR (qRT-PCR) (Figures S12 and S13). 1 µg of total RNA from all time points (24, 36, 48, 72, 120 and 168 hpi) and all three independent biological replications was transcribed into cDNA with the First Strand cDNA synthesis kit (Fermentas, St. Leon-Rot, Germany). 10 ng of cDNA was used as template for qRT-PCR using specific primers (Table S16). Primer design of all primers used in this study were based on Primer3. Specific primers for the constitutively expressed *P. indica Tef* gene [106] were used as reference gene. qRT-PCRs were performed in 20 µl iQ SYBR Green Super Mix (Bio-Rad, München, Germany) using a iCycler (Bio-Rad, München, Germany) and the following amplification protocol: initial denaturation for 10 min at 95°C, followed by 40 cycles with 30 s at 95°C, 30 s at 60°C, 30 s at 72°C and a melt curve analysis. Ct values were determined with the software supplied with the cycler. Relative expression values were calculated using the 2^−ΔΔCt^ method [107] as described previously by [22]. The absence of contaminating genomic DNA was confirmed by performing a control PCR on RNA not reverse transcribed.

### Enrichment analysis

To identify significantly enriched gene ontology (GO) terms from the microarray hybridization experiments the Gene Ontology Enrichment Analysis Software Toolkit (GOEAST) was used (http://omicslab.genetics.ac.cn/GOEAST/index.php) with settings for customized microarray platform. For the enrichment analysis the probe annotation file for gene ontology terms produced by Blast2GO was used. Induced genes during symbiosis or during growth on autoclaved root material were analyzed using the recommended parameter settings. A table summarizing all enriched GO terms was prepared from the GOEAST output and is shown in Table S11.

### Microscopy

To visualize the papillae and the hyphal adhesion zone the carbohydrate binding lectin concanavalinA (ConA) coniugated with Alexa Fluor 633 (ConA-AF633, Molecular Probes, Karlsruhe, Germany), was used. ConA selectively binds to α-mannopyranosyl and α-glucopyranosyl residues found in various sugars, glycoproteins, and glycolipids and it is generally used to visualize glycoproteins. Barley seeds (*Hordeum vulgare* L. cv. Golden Promise) were surface sterilized as described in microarray experimental design. Three days old roots were inoculated with 3 ml of *P. indica* spore suspension (500,000 chlamydospores/ml). Incubation was performed in a Conviron phytochamber (8 h 18°C dark, 16 h 22°C light). Two, three, four, five, seven and ten days post inoculation the second cm of the roots below the seed (differentiation zone) was excised and stained by infiltration (two times 4 minutes at 260 mbar) with ConA-AF633 and wheat germ agglutinin (WGA) Alexa Fluor 488 conjugate (WGA-AF488, Molecular Probes, Karlsruhe, Germany) each 10 µg/ml in 1x PBS buffer. 6×1 cm root fragments of independent biological material were analyzed for the presence of ConA stained papillae. Counting of papillae was performed by confocal microscopy (TCS-SP5 confocal microscope, Leica, Bensheim, Germany). Excitation of ConA-AF633 was done at 633 nm and detection at 650–690 nm.

Root colonization and barley cortex cells viability were analyzed by confocal microscopy. Colonized roots were stained by infiltration for 10 min with 10 µg/ml WGA-AF488 to visualize fungal structures and 1 µg/ml propidium iodide (Sigma) for plant cells in PBS buffer. Membranes were stained with 3 µM FM4–64 (Molecular Probes, Karlsruhe, Germany) for 5 min. For imaging of living cells with fluorescein diacetate (FDA, Sigma) roots were incubated for 15 min in 1 µg/ml FDA. Root samples were imaged with a TCS-SP5 confocal microscope (Leica, Bensheim, Germany) using an excitation at 488 nm for WGA-AF488 and FDA and detection at 500–540 nm. propidium iodide and FM4-64 were excited at 561 nm and detected at 580–660 nm.

To determine the nuclear ploidy level of *P. indica*, chlamydospores were collected from 4-week-old CM-agar plates with 0.002% Tween water. Chlamydospores were washed 3 times with 0.002% Tween water and resuspend in 0.9% NaCl to the final concentration of 10^10^ spores/ml. The haploid *Saccharomyces cerevisiae* genotype BY4741, MATa (ACC. No. Y02321, Euroscarf, Frankfurt), and the diploid *S. cerevisiae* genotype FY1679, MATa/MATa (ACC. No. 10000D, Euroscarf, Frankfurt) were used as standards. Yeast cells were collected by centrifugation from 4 days old liquid culture, washed three times with 0.9% NaCl and resuspended in the same buffer to a final concentration of 10^10^ cells/ml. The same volume (approx. 250 µl) of *P. indica* spore-suspension and 1n or 2n *S. cerevisiae* cells suspensions were mixed together and incubated for 15 minutes in darkness on ice with 0.5 µl of Syto 9 and propidium iodide. Excess stain was removed by washing 3 times with 0.9% NaCl. Fungal spores and cells suspensions were spread onto glass slides, covered with cover glass and analyzed under confocal laser scanning microscope, Leica TCS SP2 (Leica, Bensheim, Germany). A series of optical sectioning images were taken (set manually at 0.10 µm steps) for both *P. indica* and *S. cerevisiae* after marking the area of each nucleus. Fluorescence of each section of the nucleus was measured using the software provided with the microscope (LCS, Leica Confocal Software). At least seven nuclei were measured for each fungal strain. Based on the assumption that the amount of DNA per cell is directly proportional to the fluorescence intensity [108] the DNA content of the *P. indica* nucleus was estimated by comparing the histogram mean of the fluorescence intensity with that of the *S. cerevisiae* standards.

## Supporting Information

Figure S1 *P. indica* colonization of barley (cv. Golden Promise) roots during the biotrophic phase. A) Amount of Concanavalin A (ConA-AF633) stained papillae formed in the differentiation zone (2 cm underneath the germinated seed) in response to *P. indica* colonization. Papillae from the outermost layers of barley root cortex cells were stained with the carbohydrate-binding ConA. The number of ConA-stainable papillae formed in response to *P. indica* penetration attempts raises during the early biotrophic phase (2 to 5 dpi) and decreases at the late biotrophic phase (7 to 8 dpi), eventually reaching zero at the cell death-associated phase (from 10 dpi onwards). Error bars were calculated as standard error of the mean. At least 6 plants grown on 1/10 PNM medium were used at each time point. B) Relative amount of fungal DNA in colonized barley roots grown on 1/10 PNM medium at different time points (2, 3, 5 and 7 dpi). Three biological repetitions were performed showing a similar fungal colonization profile. This material was subsequently used for the microarrays hybridization and qPCR analyses. C) Amount of fluorescein diacetate (FDA) stained cells from the outermost layers of barley root cortex cells in *P. indica* colonized (white bars) and non colonized (black bars) roots. Plants were grown on 1/10 PNM medium. FDA is non fluorescent, but when hydrolyzed by intracellular esterases, the hydrophilic fluorescent product fluorescein is formed indicative of cell viability. The reduction in number of vital cells from 4 to 7 dpi is most likely due to an early natural senescence process characteristic for barley and other cereals, called root cortical cell death (RCD). In barley the onset of the apoptotic process starts about two days after seed germination and became more pronounced in older root segments [114], [115]. Root colonization by *P. indica* did not significantly influence root cortical cell death (FDA staining) at 4 and 7 dpi (biotrophic phase) in the outermost layers. Error bars were calculated as standard error of the mean of 4 biological replicates. D) Schematic representation of *P. indica* colonization of the differentiation zone from barley roots at 4 dpi (7 days old roots). At this stage a mixture of colonized vital (red) and non vital (black) cells is present. Living cells are intracellularly colonized by a single hyphae with no or limited branching, whereas dead cells are extensively colonized.
(TIF)

Figure S2 Bar charts show the top 10 organisms with best blast hits (cut off eVal 10^−3^) for either *P. indica* transcriptome (11769, left), secretome (867, right – whole bars) or for the secreted proteins that are less than 300 aa in size (366, right – black bars). Blast searches were performed with Blast2GO [94]. Diagrams were created using gnuplot (version 4.4 patchlevel 2; Williams and Kelley; www.gnuplot.info).
(TIF)

Figure S3 Conserved syntenic gene blocks. Diagram representing *P. indica* syntenic gene blocks conserved in *L. bicolor v2.0* (88), *C. cinerea* (49), and *U. maydis* (10). Each block consists of at least 2 adjacent genes displaying substantial similarity and conserved gene order between the related fungi. The analyses were performed using: (1) bidirectional best blastp hits with an e value ≤1e^−19^ and alignment length >75% of the query protein length or (2) bidirectional best blastp hits with an e value ≤1e^−19^ and similar definition line annotation as judged manually excluding hypothetical proteins or (3) genes with exactly the same definition line annotation excluding hypothetical proteins.
(TIF)

Figure S4 Nucleotide preference at each codon position from 20 different fungi. The codon usage of *A. bisporus* (Abiva), *A. nidulans* (Asni), *C. cinerea* (Coci), *C. neoformans* (Cryne), *F. oxysporum* (Fusox), *H. annosum* (Heta), *L. bicolor* (Labi), *M. populina* (Melapo), *P. crysoporium* (Phac), *P. indica* (Piri), *P. ostreatus* (Pleos), *P. placenta* (Popl), *P. graminis* (Pugr), *S. commune* (Schico), *S. lacrymans* (Serla), *S. roseus* (Sporo), *T. atroviride* (Trat), *T. mesenterica* (Treme), *T. reesei* (Trire) and *U. maydis* (Usti) was calculated using JAVA. The output was used to create frequency plots by WebLogo [84].
(TIF)

Figure S5 Representation of the putative *MAT-A* region from *P. indica* containing the multiallelic homeodomain encoding genes of the two classes of DNA binding motifs (HD1 and HD2, gray arrows). Best hit for PIIN_09915 is the A1 mating-type protein from *P. chrysosporium* (e value, 1e^−03^). Best hit for PIIN_09916 is the A2 mating-type protein from *P. chrysosporium* (e value, 1e^−06^). Average coverage for *P. indica* contigs 0565 and 0582 was 13.27 and 8.58 respectively. No SNPs were found. ESTs from RNA-Seq of cDNA pooled from various *P. indica* developing stages matched the putative HD1.1 and HD1.2. The white arrows indicate hypothetical ORFs predicted from the automated annotation pipeline. No conserved domains were identified in these proteins. Best hit for PIIN_09914 is PIIN_09976 with an e value of 0.0.
(TIF)

Figure S6 Measurement of fluorescence intensity of *Saccharomyces cerevisiae* and *Piriformospora indica* nuclei. To determine ploidy level, fungal nuclei were stained with the DNA intercalating dye syto9. Based on the assumption that the amount of DNA per cell is directly proportional to the fluorescence intensity [108] the DNA content of the *P. indica* nucleus was estimated by comparing the histogram mean of optical sections with those of the *S. cerevisiae* standards. Based on the genome size estimation from pyrosequencing (24.98 Mb), the nuclear fluorescence intensity suggest a ploidy level of 1n for *P. indica*. Together with single nucleotide polymorphism (SNPs) analysis this indicates that the *P. indica* strain sequenced is an heterokaryon. Histogram mean of optical sections was calculated with the LCS, Leica Confocal Software on a TCS SP5 CLM (Leica, Bensheim, Germany).
(TIF)

Figure S7 The upper panel show a scatterplot of the average coverage of Paired End (PE) contigs vs contig length. Three groups of contigs could be clustered based on the average coverage (200, 22 and 10). The overall average coverage of the contigs was 21.74, but the plot shows that smaller contigs can differ significantly from this average. Contigs with low coverage ranging from 8 to 14 had predominately no SNPs. These highly polymorphic regions in the genome of *P. indica* could neither be assembled nor assigned to a specific chromosome. Contigs with an average coverage of 200 could be assigned to the mitochondrion of *P. indica*. The high coverage is typical for these sequences. The plot was created using gnuplot (version 4.4 patchlevel 2). The lower panel show a scatterplot of the GC-content of Paired End (PE) contigs vs contig length. Two groups of contigs could be identified by GC content analysis. The overall average GC-content of the contigs was calculated to be 52.3%. The contigs that could be assigned to the mitochondrion of *P. indica* had a lower GC-content which is typical for these sequences. No additional significant digression from the 52.3% average was found. The plot was created using gnuplot (version 4.4 patchlevel 2; Williams and Kelley; www.gnuplot.info).
(TIF)

Figure S8 Distribution of single nucleotide polymorphisms (SNPs) in *P. indica* contigs (left). The plot shows averaged SNPs calls/kb versus averaged coverage/contigs with an interval of 1. 1.87 Mb of the *P. indica* genome is represented by low coverage (<12) contigs containing almost no SNPs (total number of SNPs: 347; 0.18 SNPs/kb) while 22.98 Mb of the genome is represented by high coverage (12–26) contigs containing most of the SNPs (total number of SNPs: 6,0079; 2.61 SNPs/kb). Scatterplot of total number of SNPs called per contig versus contig length (right). The plot shows that there is a linear correlation between the number of SNPs and the contig length (R^2^ = 0.86). Both plots were created using gnuplot (version 4.4 patchlevel 2; Williams and Kelley; www.gnuplot.info).
(TIF)

Figure S9 Protein families in *P. indica* and related organisms. a, Number of protein families compared against genome size (number of predicted ORFs) (blue). b, Average number of proteins per protein family compared against genome size (orange). Clustering of protein families was performed using the Tribe-MCL algorithm [41] as described in material and methods.
(TIF)

Figure S10 Nitrogen assimilation test. *P. indica* was grown on Yeast Nitrogen Base (YNB) agar medium without amino acids and ammonium sulfate (DIFCO, REF 233520). 20% glucose was used as C source. The medium was buffered with 0.1 M KH_2_PO_4_-K_2_HPO_4_ buffers. The final pH of the cultures varied little from the initial pH 7. Plates either contained no nitrogen, or were supplemented with N in the form of 0.5 mM and 2 mM nitrate (KNO_3_), 0.5 mM and 2 mM ammonium (NH_4_Cl), or 0.25 mM and 1 mM glutamine (Gln). Plates were inoculated with the same amount of chlamydospores (500,000/ml) and analyzed after 5 days. *P. indica* growth on nitrate was comparable to the growth on the control medium without N source. Ammonium as N source provided the greatest growth ratio, followed by glutamine treatments. These results are consistent with the genome wide analyses that inferred the absence of nitrate transporters, nitrate and nitrite reductases.
(TIF)

Figure S11 Sequence logos from hidden markov models (HMM) created using LogoMat-P [116]. HMMs were created using HMMER (version 3.0, http://hmmer.org/) based on multiple sequence alignments constructed using MUSCLE [112]. a) The LysM model was created using 61 *P. indica* LysM domains (from 18 proteins) and compared to the LysM model from the Pfam database [38]. The generated HMM logo shows that *P. indica* LysM domains contain 3 conserved cysteine residues at positions 9, 32 and 42 as described for *Tricoderma atroviride* [117]. b) The WSC model was created using 109 *P. indica* WSC domains (from 36 proteins) and compared to the WSC model from the Pfam database. c) The CBM1 model was created using 69 *P. indica* LysM domains (from 67 proteins) and compared to the CBM1 model from the Pfam database. Used were all *P. indica* domains which were classified as CBM1 by the Pfam database and as fCBD by SMART [101]. The constructed *P. indica* CBM1 model was further compared to all other CBM models from the Pfam database. LogoMat-P produced the best alignment for CBM1 and only small alignments of the HMMs for all other CBM domains indicating that the 69 domains identified in *P. indica* belong to the CBM1 category.
(TIF)

Figure S12 Mean fold-change estimates for selected *P. indica* genes for comparison between microarray and quantitative PCR methods. Fold changes were determined for *P. indica* growing on living (blue) or dead (red) barley roots by the 2^−ΔCt^ method [118] and calculated relative to complete medium (CM) control. Expression data are standardized relative to PiTEF. Standard errors are from 3 independent biological repetitions.
(TIF)

Figure S13 Quantitative PCR analysis of plant (left) and fungal (right) transporters involved in different forms of nitrogen uptake. The plant and fungal transporters were up-regulated upon *P. indica* colonization of barley roots grown on plant minimal medium (PNM) in axenic condition. A) Relative expression of two putative nitrate transporters (Hv NRT1, Harvest Unigene 46286 and 39899) and one ammonium transporter (Hv AMT, 10619) from barley in response to *P. indica* colonization at 1, 2, 3 and 5 dpi. Fold changes were determined by the 2^−ΔCt^ method [118] and were calculated relative to non inoculated barley roots control. Expression data are calculated relative to barley ubiquitin (M60175). B) Relative expression of two *P. indica* ammonium transporters (PiAMT1, PIIN_02036; PiAMT2, PIIN_04373) during colonization of living barley roots. Fold changes were determined by the 2^−ΔCt^ method and were calculated relative to *P. indica* TEF (AJ249911, PIIN_03008) versus the control complete medium. Relative expression values of selected transcripts were similar in 3 independent biological experiments.
(TIF)

Figure S14 Alignment of the central part of *Piriformospora indica* DELD proteins and HRPII proteins from *Plasmodium falciparum*. Two representative DELD proteins and HRPII proteins were chosen for the alignment. While looking for DELD homologs from other organisms, we found that HRPII, a protein synthesized by the parasite during the early erythrocyte infection, shows about 30% sequence identity with the central part of the DELD proteins, primarily due to its high histidine and alanine content.
(TIF)

Figure S15 Distribution of *P. indica* intergenic region lengths. 9010 predicted genes were sorted into two dimensional bins on the basis of the lengths of the flanking intergenic distances to neighboring genes at the 5′ and 3′ ends as described before [119]. The number of genes in each bin is shown as a log transformed color-coded (z axis) heat map. 2759 genes either present alone or at the end of the scaffolds hence lacking neighboring genes were excluded from the analysis. *P. indica* genome does not show an unusual distribution of intergenic region lengths but possess a gene dense genome with an average distance between genes of 530 bp (see also Table S2). 279 genes were present in gene-poor regions with intergenic space between 1 kb and 9 kb. From the 279 genes, 43 (15.41%) were predicted to be secreted and of these, 18 (41.86%) were differentially regulated during colonization of barley roots. Additionally, similar to effectors found in other filamentous organisms, genes belonging to the *P. indica* putative effector family DELD proved to have flanking intergenic distances among the longest (with an average at the 5′ of 1677 bp and at the 3′ of 1345 bp).
(TIF)

Figure S16 Circular map of the *P. indica* mitochondrion. The map was drawn using CGView [120]. Different colours indicate different gene families: blue: cytochrome c oxidase (subunits COX1, COX2, COX3) and cytochrome b (COB); red: NADH dehydrogenases (subunits NAD1, NAD2, NAD3, NAD4, NAD4L, NAD5, NAD6); green: ATPases (subunits ATP6, ATP8, ATP9); grey: subunits of the ribosome (SSUrna, LSUrna) and ribosomal proteins (rps3); gold: transfer RNAs (V = Valine, P = Proline, N = Asparagine, L = Leucine, R = Arginine, G = Glycine, M = Methionine, E = Glutamate, T = Threonine, Q = Glutamine, K = Lysine, C = Cysteine, A = Alanine, F = Phenylalanine, Y = Tyrosine, S = Serine, I = Isoleucine, H = Histidine, D = Aspartate, W = Tryptophan), numbers indicate multiple copies of the tRNA, asterisk indicate confirmation only by one program (tRNAscan-SE, [83], or Aragorn, [121]); dark blue: homing endonucleases with LAGLIDADG (LAG) or GIY-YIG (GIY) motif; introns are drawn in the same colour as their corresponding genes, but a bit lighter; numbers indicate the position and number of introns in the specific gene (In1–In4).
(TIF)

Table S1 Evaluation of *P. indica* annotation against mapped ESTs.
(XLS)

Table S2 Genome features of *P. indica* and related fungi.
(XLS)

Table S3 Repetitive DNA sequences identified in the *P. indica* genome.
(XLS)

Table S4 tRNA genes along with their respective anticodons present in *P. indica.*
(XLS)

Table S5 Single nucleotide polymorphisms identified in the *P. indica* contigs.
(XLS)

Table S6 Clustering of protein domains.
(XLS)

Table S7 Structural comparison of LysM and WSC proteins in different fungi.
(XLS)

Table S8 Clustering of protein families.
(XLS)

Table S9 Analyses of genes and clusters involved in secondary metabolite production.
(XLS)

Table S10 Significantly regulated genes from microarray data and selected features.
(XLS)

Table S11 Enrichment analysis performed using GOEAST.
(XLS)

Table S12 Identified RSIDELD motif in fungi.
(XLS)

Table S13 Identified RXLR motifs in *P. indica.*
(XLS)

Table S14 Reference genomes.
(XLS)

Table S15 Subcellular localization of *P. indica* proteins.
(XLS)

Table S16 List of primers used in this study.
(XLS)

Table S17 EMBL accession numbers.
(XLSX)
