# Supplementary figures and images for: Endophytic Life Strategies Decoded by Genome and Transcriptome Analyses of the Mutualistic Root Symbiont Piriformospora indica
Source: PLoS Pathog. 2011 Oct 13;7(10):e1002290. doi: 10.1371/journal.ppat.1002290 (PMC3192844; doi:10.1371/journal.ppat.1002290)

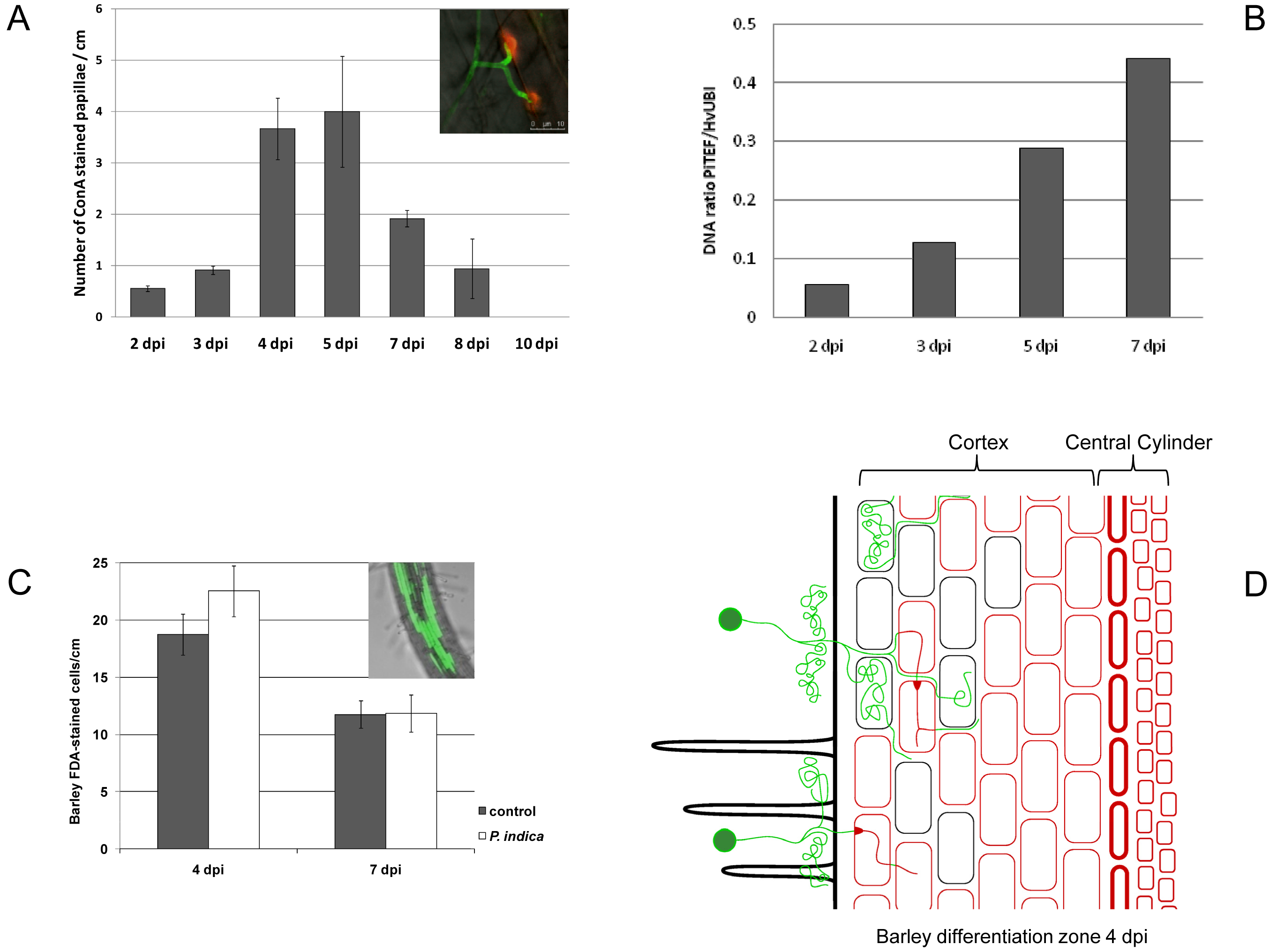

Supplement: Figure S1 — P. indica colonization of barley (cv. Golden Promise) roots during the biotrophic phase. A) Amount of Concanavalin A (ConA-AF633) stained papillae formed in the differentiation zone (2 cm underneath the germinated seed) in response to P. indica colonization. Papillae from the outermost layers of barley root cortex cells were stained with the carbohydrate-binding ConA. The number of ConA-stainable papillae formed in response to P. indica penetration attempts raises during the early biotrophic phase (2 to 5 dpi) and decreases at the late biotrophic phase (7 to 8 dpi), eventually reaching zero at the cell death-associated phase (from 10 dpi onwards). Error bars were calculated as standard error of the mean. At least 6 plants grown on 1/10 PNM medium were used at each time point. B) Relative amount of fungal DNA in colonized barley roots grown on 1/10 PNM medium at different time points (2, 3, 5 and 7 dpi). Three biological repetitions were performed showing a similar fungal colonization profile. This material was subsequently used for the microarrays hybridization and qPCR analyses. C) Amount of fluorescein diacetate (FDA) stained cells from the outermost layers of barley root cortex cells in P. indica colonized (white bars) and non colonized (black bars) roots. Plants were grown on 1/10 PNM medium. FDA is non fluorescent, but when hydrolyzed by intracellular esterases, the hydrophilic fluorescent product fluorescein is formed indicative of cell viability. The reduction in number of vital cells from 4 to 7 dpi is most likely due to an early natural senescence process characteristic for barley and other cereals, called root cortical cell death (RCD). In barley the onset of the apoptotic process starts about two days after seed germination and became more pronounced in older root segments [114], [115]. Root colonization by P. indica did not significantly influence root cortical cell death (FDA staining) at 4 and 7 dpi (biotrophic phase) in the outermost layers. Error bar [file ppat.1002290.s001.tif]

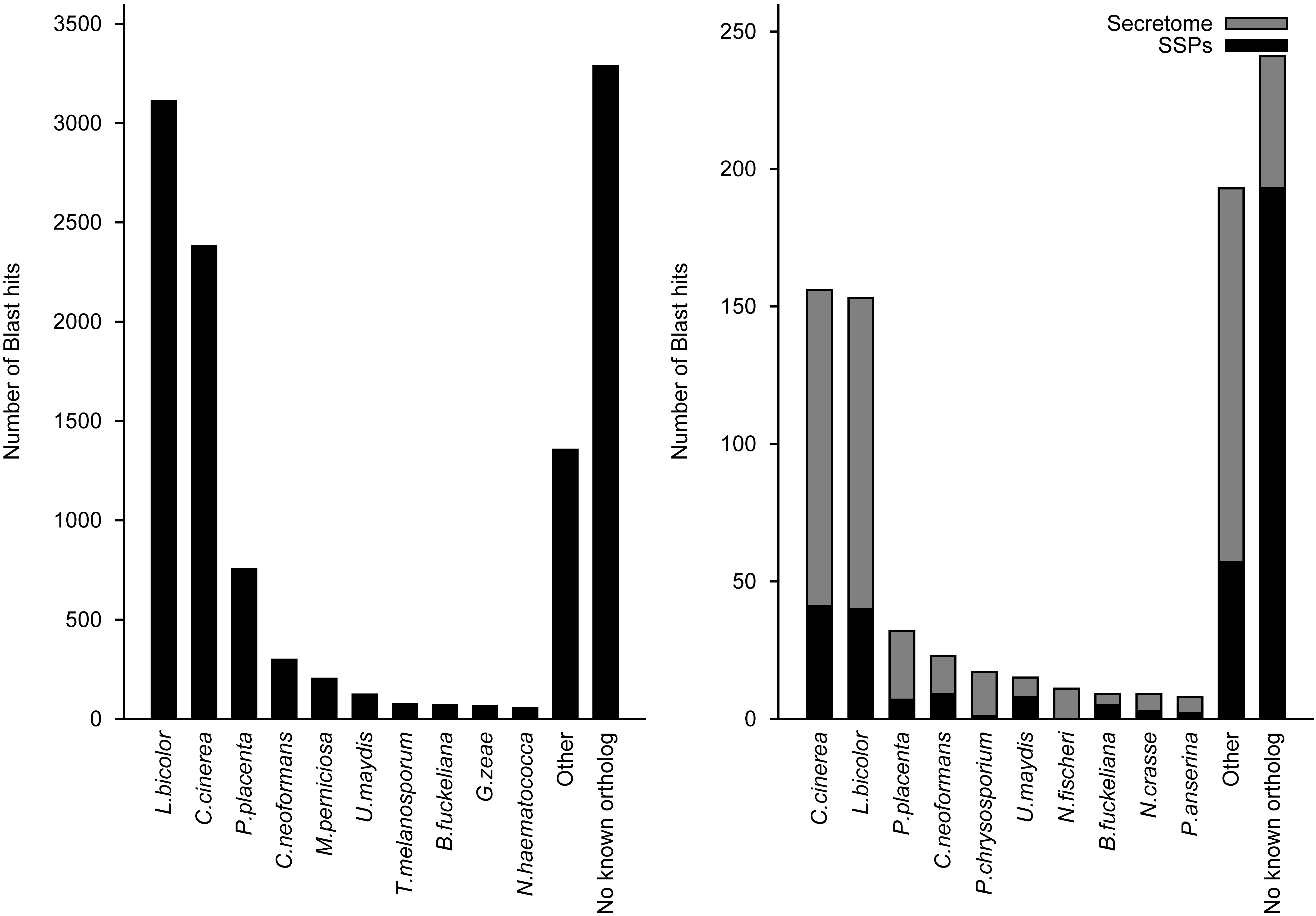

Supplement: Figure S2 — Bar charts show the top 10 organisms with best blast hits (cut off eVal 10−3) for either P. indica transcriptome (11769, left), secretome (867, right – whole bars) or for the secreted proteins that are less than 300 aa in size (366, right – black bars). Blast searches were performed with Blast2GO [94]. Diagrams were created using gnuplot (version 4.4 patchlevel 2; Williams and Kelley; www.gnuplot.info). (TIF) [file ppat.1002290.s002.tif]

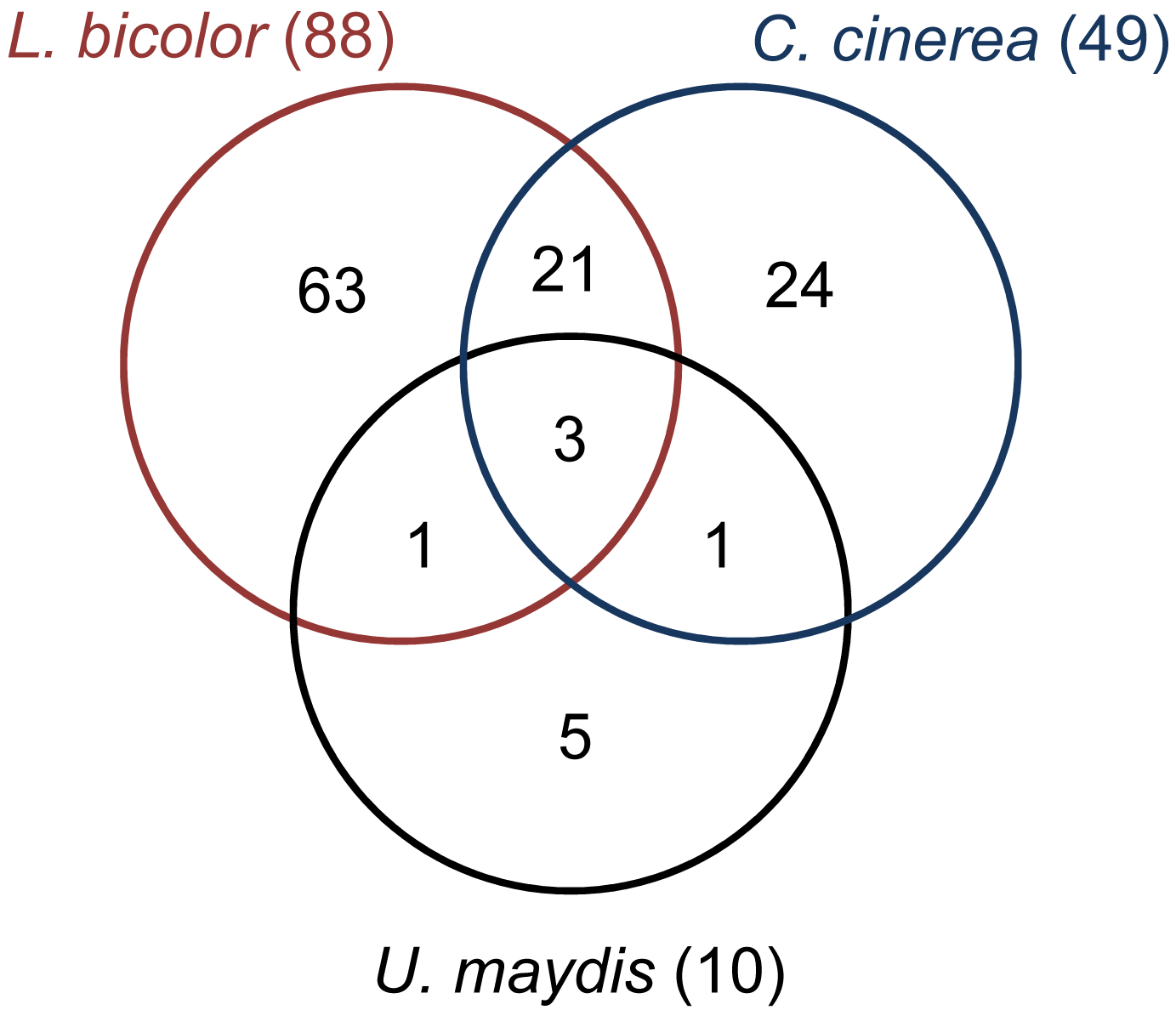

Supplement: Figure S3 — Conserved syntenic gene blocks. Diagram representing P. indica syntenic gene blocks conserved in L. bicolor v2.0 (88), C. cinerea (49), and U. maydis (10). Each block consists of at least 2 adjacent genes displaying substantial similarity and conserved gene order between the related fungi. The analyses were performed using: (1) bidirectional best blastp hits with an e value ≤1e−19 and alignment length >75% of the query protein length or (2) bidirectional best blastp hits with an e value ≤1e−19 and similar definition line annotation as judged manually excluding hypothetical proteins or (3) genes with exactly the same definition line annotation excluding hypothetical proteins. (TIF) [file ppat.1002290.s003.tif]

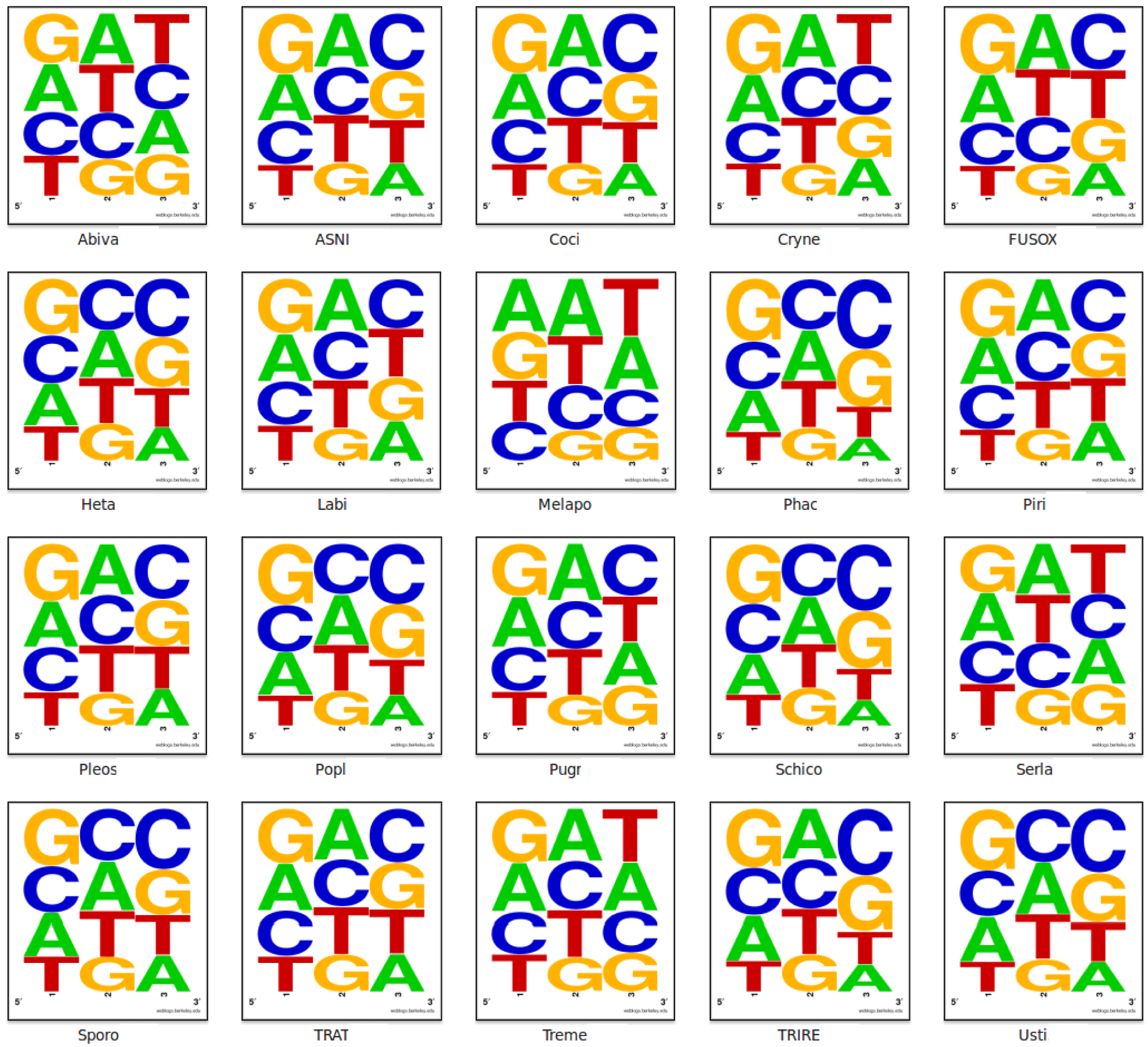

Supplement: Figure S4 — Nucleotide preference at each codon position from 20 different fungi. The codon usage of A. bisporus (Abiva), A. nidulans (Asni), C. cinerea (Coci), C. neoformans (Cryne), F. oxysporum (Fusox), H. annosum (Heta), L. bicolor (Labi), M. populina (Melapo), P. crysoporium (Phac), P. indica (Piri), P. ostreatus (Pleos), P. placenta (Popl), P. graminis (Pugr), S. commune (Schico), S. lacrymans (Serla), S. roseus (Sporo), T. atroviride (Trat), T. mesenterica (Treme), T. reesei (Trire) and U. maydis (Usti) was calculated using JAVA. The output was used to create frequency plots by WebLogo [84]. (TIF) [file ppat.1002290.s004.tif]

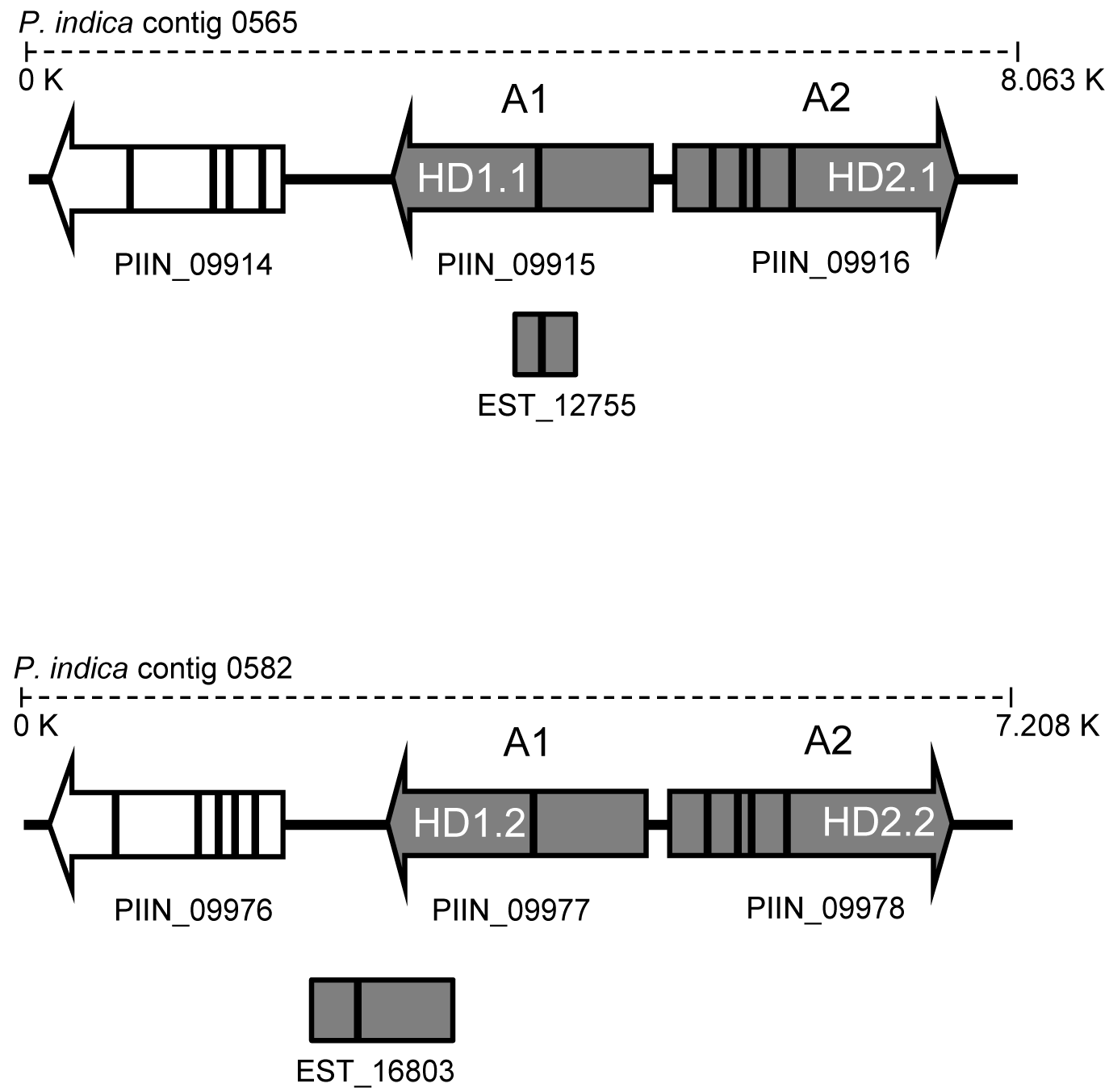

Supplement: Figure S5 — Representation of the putative MAT-A region from P. indica containing the multiallelic homeodomain encoding genes of the two classes of DNA binding motifs (HD1 and HD2, gray arrows). Best hit for PIIN_09915 is the A1 mating-type protein from P. chrysosporium (e value, 1e−03). Best hit for PIIN_09916 is the A2 mating-type protein from P. chrysosporium (e value, 1e−06). Average coverage for P. indica contigs 0565 and 0582 was 13.27 and 8.58 respectively. No SNPs were found. ESTs from RNA-Seq of cDNA pooled from various P. indica developing stages matched the putative HD1.1 and HD1.2. The white arrows indicate hypothetical ORFs predicted from the automated annotation pipeline. No conserved domains were identified in these proteins. Best hit for PIIN_09914 is PIIN_09976 with an e value of 0.0. (TIF) [file ppat.1002290.s005.tif]

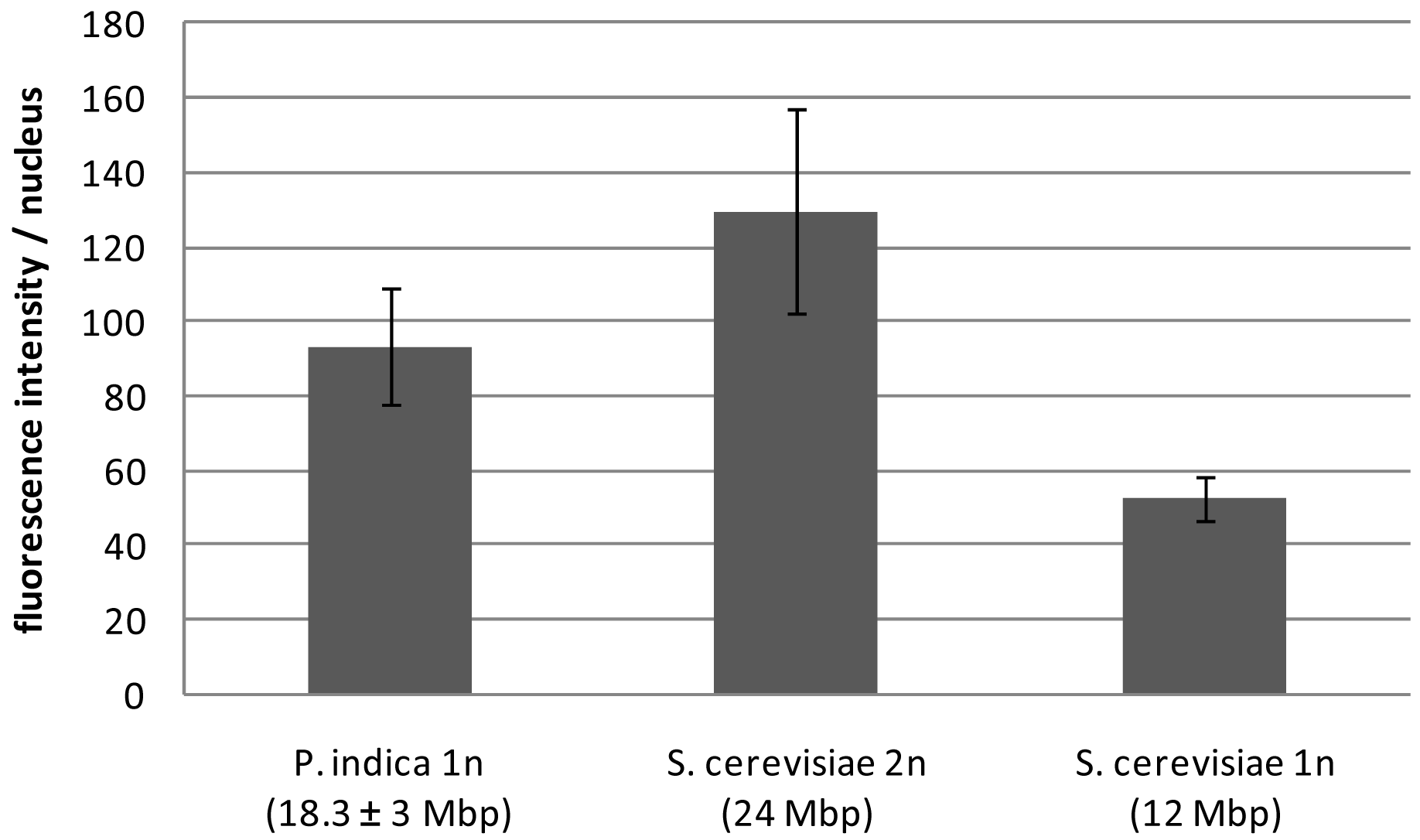

Supplement: Figure S6 — Measurement of fluorescence intensity of Saccharomyces cerevisiae and Piriformospora indica nuclei. To determine ploidy level, fungal nuclei were stained with the DNA intercalating dye syto9. Based on the assumption that the amount of DNA per cell is directly proportional to the fluorescence intensity [108] the DNA content of the P. indica nucleus was estimated by comparing the histogram mean of optical sections with those of the S. cerevisiae standards. Based on the genome size estimation from pyrosequencing (24.98 Mb), the nuclear fluorescence intensity suggest a ploidy level of 1n for P. indica. Together with single nucleotide polymorphism (SNPs) analysis this indicates that the P. indica strain sequenced is an heterokaryon. Histogram mean of optical sections was calculated with the LCS, Leica Confocal Software on a TCS SP5 CLM (Leica, Bensheim, Germany). (TIF) [file ppat.1002290.s006.tif]

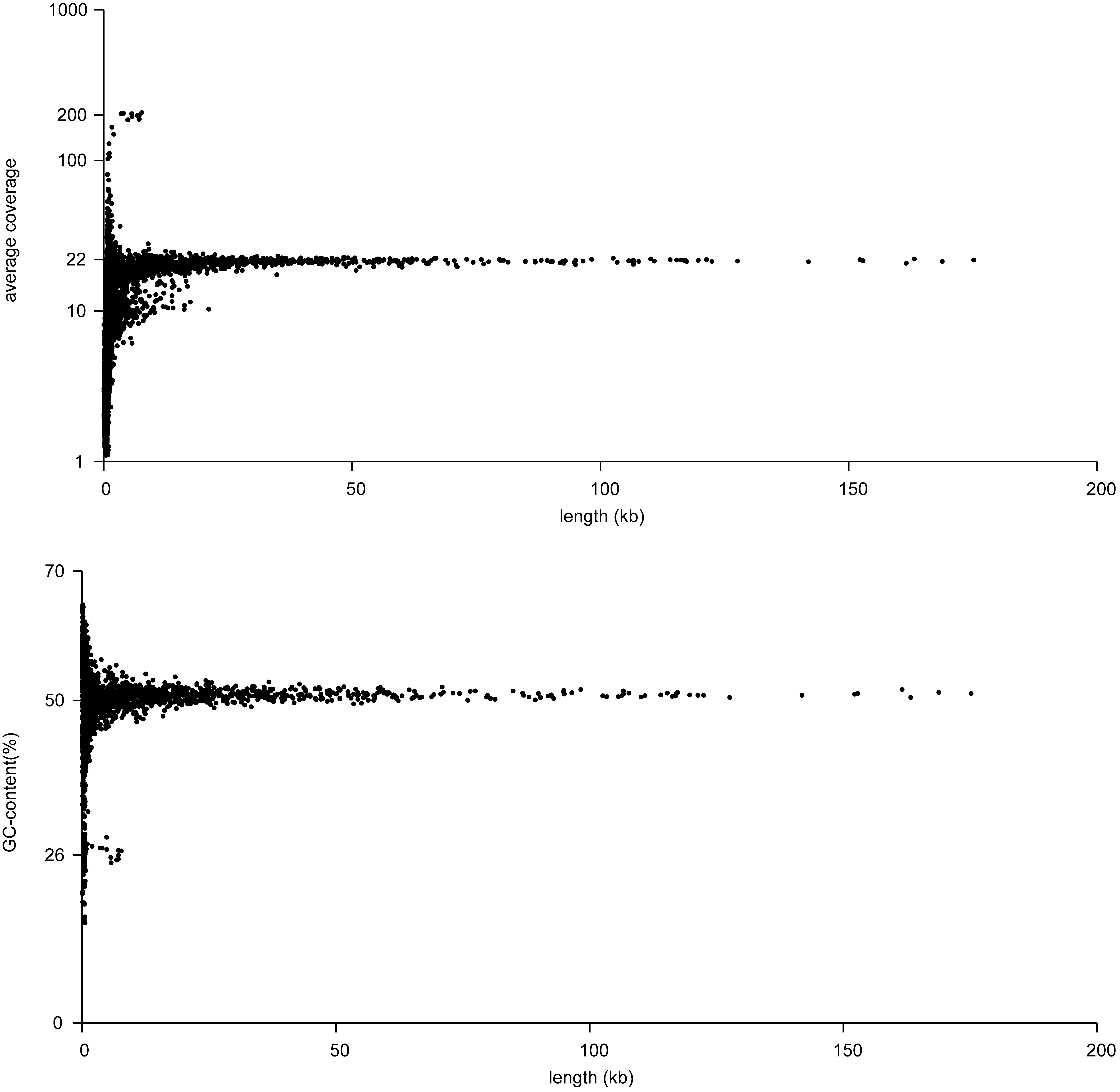

Supplement: Figure S7 — The upper panel show a scatterplot of the average coverage of Paired End (PE) contigs vs contig length. Three groups of contigs could be clustered based on the average coverage (200, 22 and 10). The overall average coverage of the contigs was 21.74, but the plot shows that smaller contigs can differ significantly from this average. Contigs with low coverage ranging from 8 to 14 had predominately no SNPs. These highly polymorphic regions in the genome of P. indica could neither be assembled nor assigned to a specific chromosome. Contigs with an average coverage of 200 could be assigned to the mitochondrion of P. indica. The high coverage is typical for these sequences. The plot was created using gnuplot (version 4.4 patchlevel 2). The lower panel show a scatterplot of the GC-content of Paired End (PE) contigs vs contig length. Two groups of contigs could be identified by GC content analysis. The overall average GC-content of the contigs was calculated to be 52.3%. The contigs that could be assigned to the mitochondrion of P. indica had a lower GC-content which is typical for these sequences. No additional significant digression from the 52.3% average was found. The plot was created using gnuplot (version 4.4 patchlevel 2; Williams and Kelley; www.gnuplot.info). (TIF) [file ppat.1002290.s007.tif]

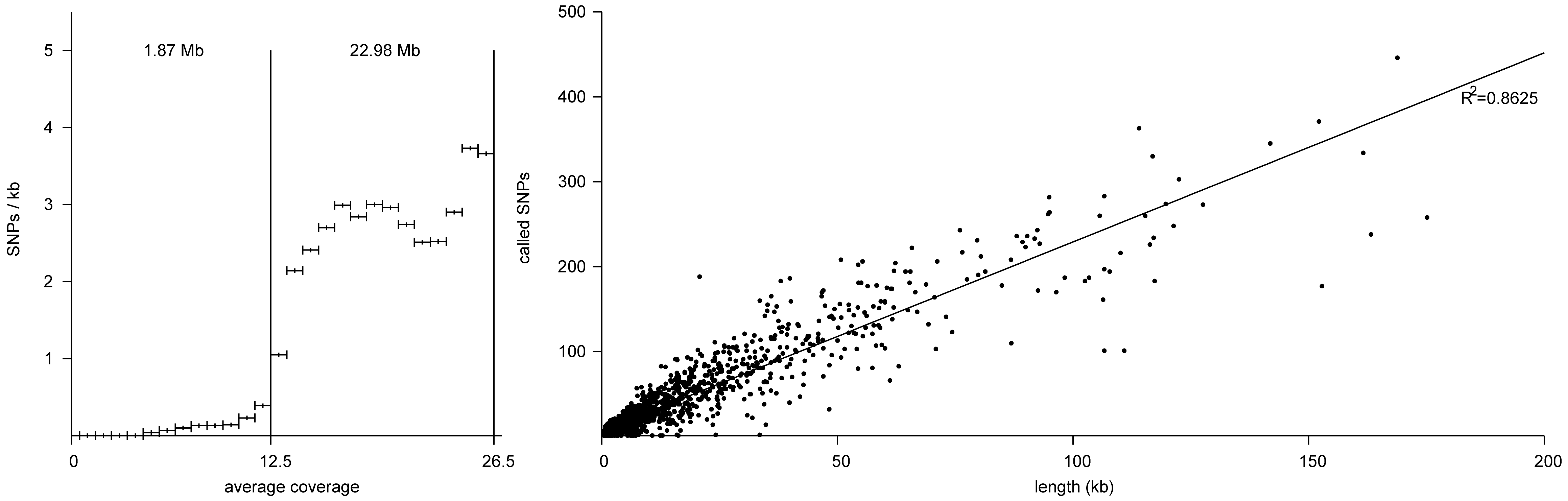

Supplement: Figure S8 — Distribution of single nucleotide polymorphisms (SNPs) in P. indica contigs (left). The plot shows averaged SNPs calls/kb versus averaged coverage/contigs with an interval of 1. 1.87 Mb of the P. indica genome is represented by low coverage (<12) contigs containing almost no SNPs (total number of SNPs: 347; 0.18 SNPs/kb) while 22.98 Mb of the genome is represented by high coverage (12–26) contigs containing most of the SNPs (total number of SNPs: 6,0079; 2.61 SNPs/kb). Scatterplot of total number of SNPs called per contig versus contig length (right). The plot shows that there is a linear correlation between the number of SNPs and the contig length (R2 = 0.86). Both plots were created using gnuplot (version 4.4 patchlevel 2; Williams and Kelley; www.gnuplot.info). (TIF) [file ppat.1002290.s008.tif]

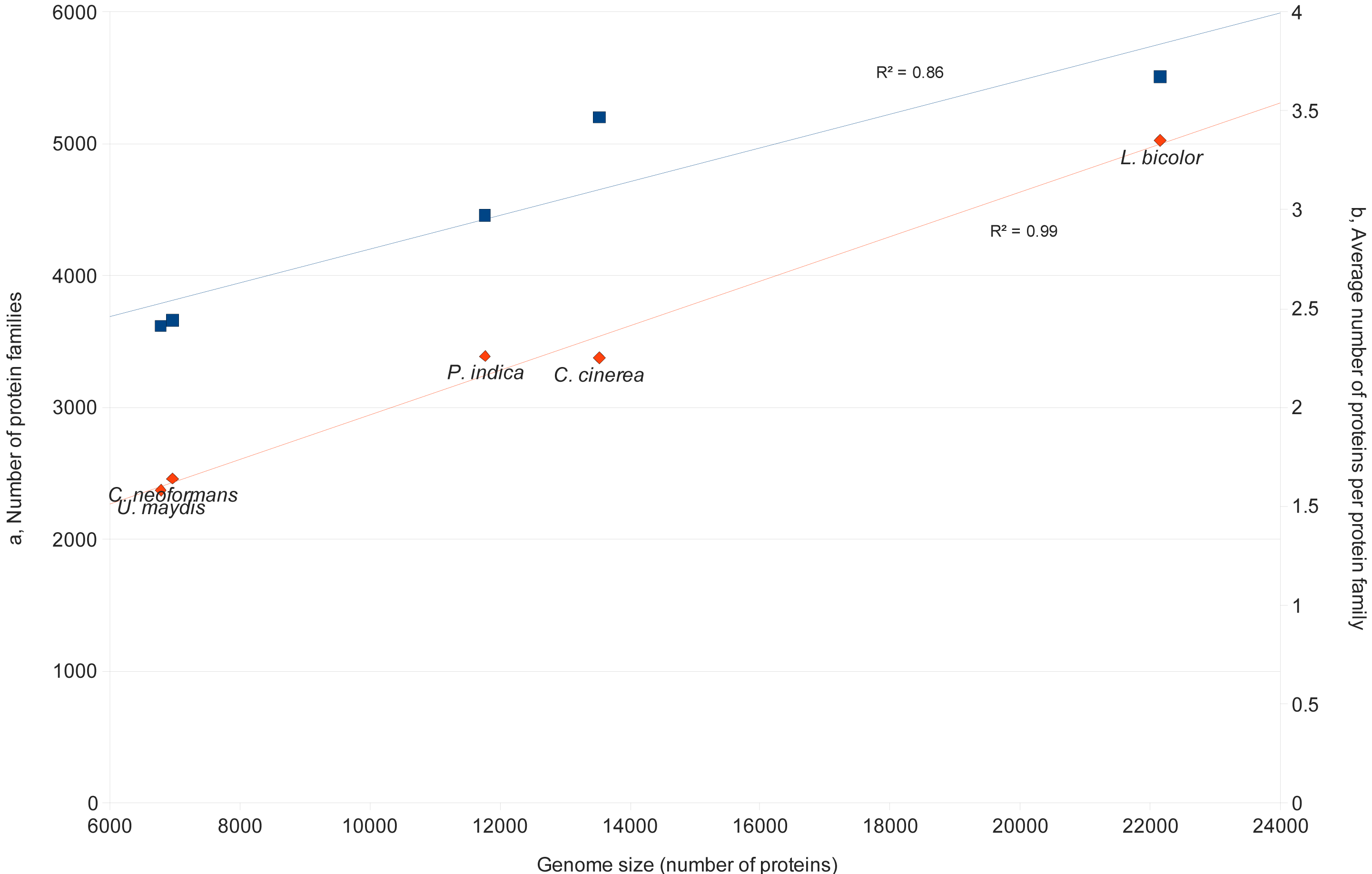

Supplement: Figure S9 — Protein families in P. indica and related organisms. a, Number of protein families compared against genome size (number of predicted ORFs) (blue). b, Average number of proteins per protein family compared against genome size (orange). Clustering of protein families was performed using the Tribe-MCL algorithm [41] as described in material and methods. (TIF) [file ppat.1002290.s009.tif]

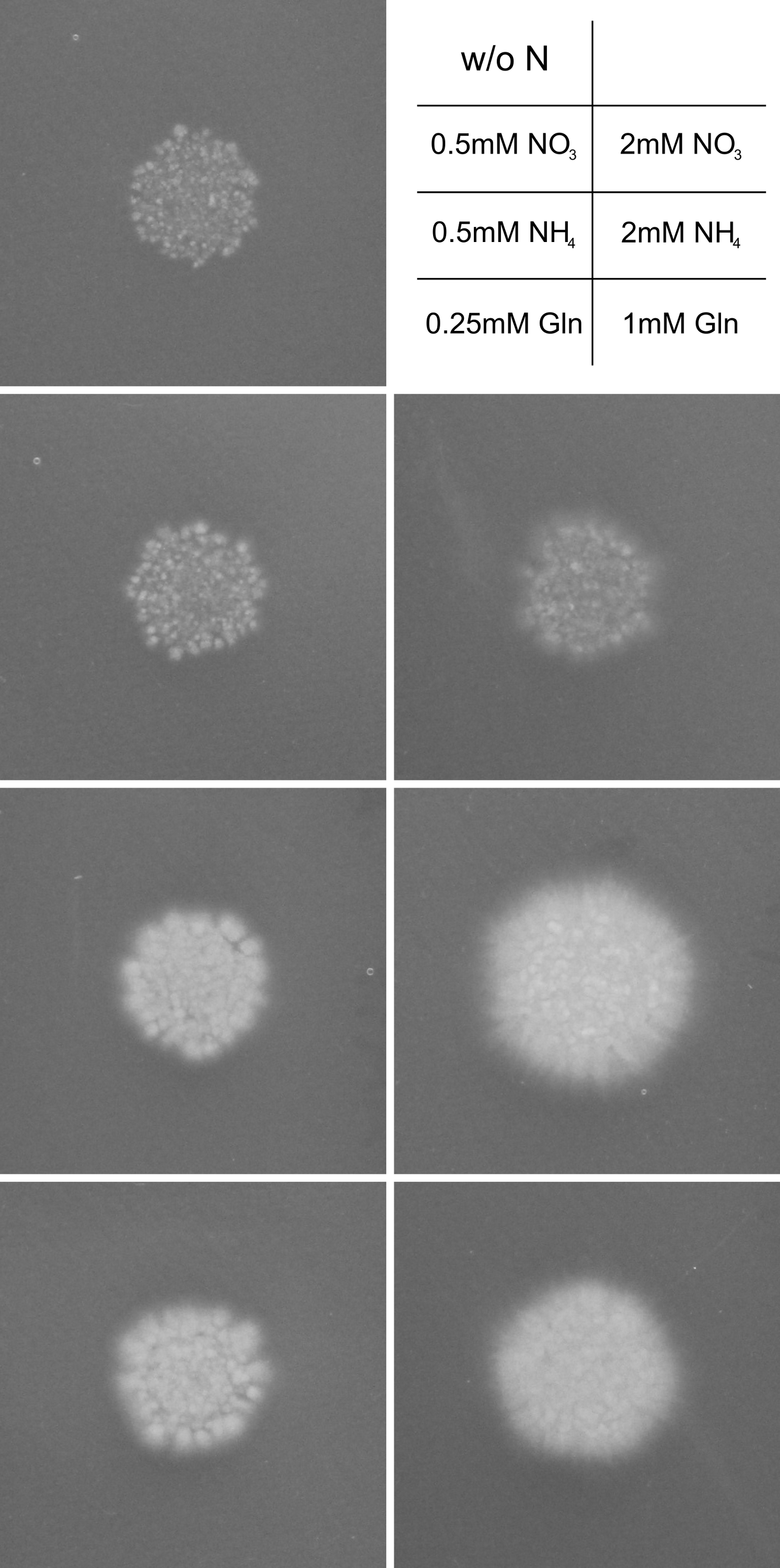

Supplement: Figure S10 — Nitrogen assimilation test. P. indica was grown on Yeast Nitrogen Base (YNB) agar medium without amino acids and ammonium sulfate (DIFCO, REF 233520). 20% glucose was used as C source. The medium was buffered with 0.1 M KH2PO4-K2HPO4 buffers. The final pH of the cultures varied little from the initial pH 7. Plates either contained no nitrogen, or were supplemented with N in the form of 0.5 mM and 2 mM nitrate (KNO3), 0.5 mM and 2 mM ammonium (NH4Cl), or 0.25 mM and 1 mM glutamine (Gln). Plates were inoculated with the same amount of chlamydospores (500,000/ml) and analyzed after 5 days. P. indica growth on nitrate was comparable to the growth on the control medium without N source. Ammonium as N source provided the greatest growth ratio, followed by glutamine treatments. These results are consistent with the genome wide analyses that inferred the absence of nitrate transporters, nitrate and nitrite reductases. (TIF) [file ppat.1002290.s010.tif]

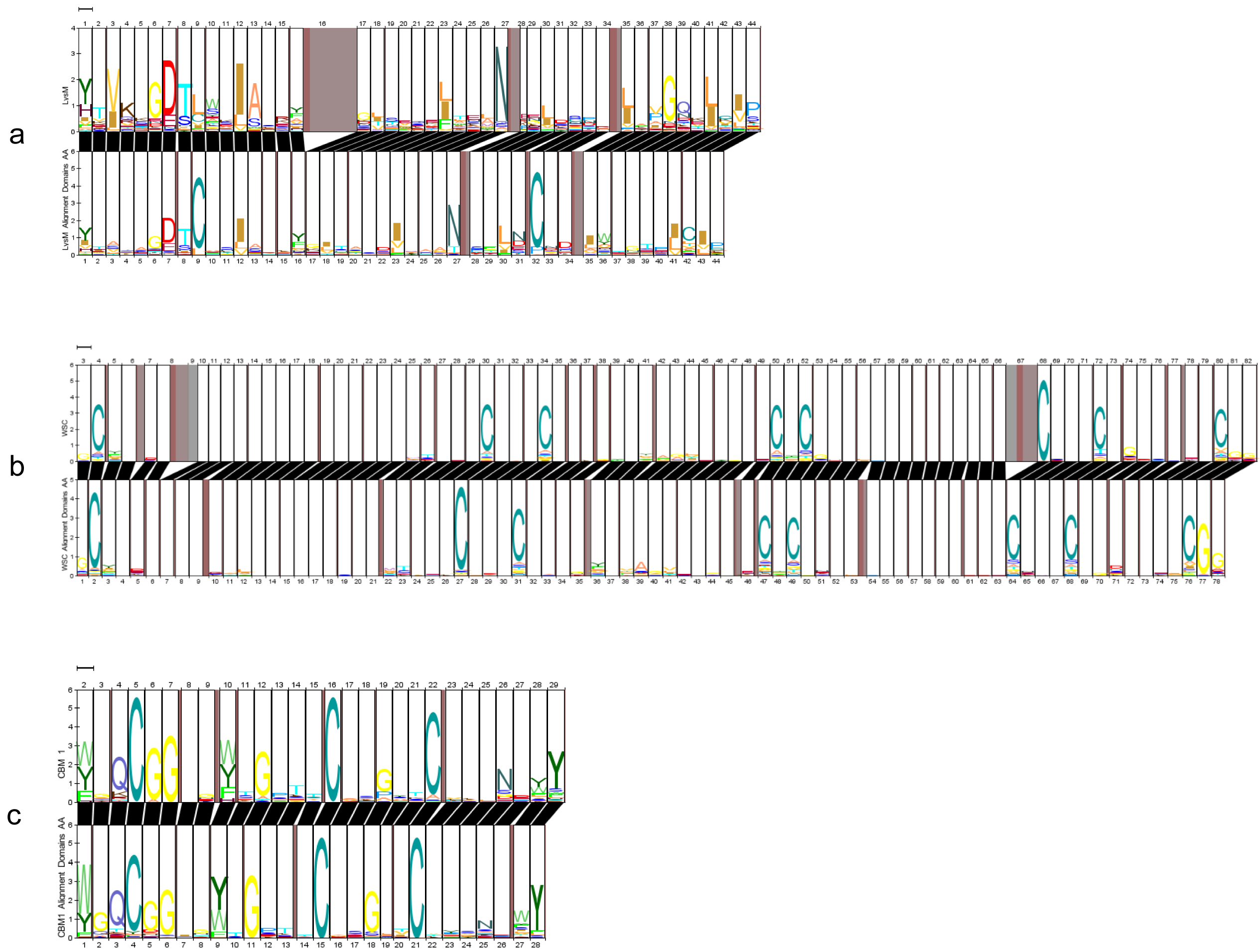

Supplement: Figure S11 — Sequence logos from hidden markov models (HMM) created using LogoMat-P [116]. HMMs were created using HMMER (version 3.0, http://hmmer.org/) based on multiple sequence alignments constructed using MUSCLE [112]. a) The LysM model was created using 61 P. indica LysM domains (from 18 proteins) and compared to the LysM model from the Pfam database [38]. The generated HMM logo shows that P. indica LysM domains contain 3 conserved cysteine residues at positions 9, 32 and 42 as described for Tricoderma atroviride [117]. b) The WSC model was created using 109 P. indica WSC domains (from 36 proteins) and compared to the WSC model from the Pfam database. c) The CBM1 model was created using 69 P. indica LysM domains (from 67 proteins) and compared to the CBM1 model from the Pfam database. Used were all P. indica domains which were classified as CBM1 by the Pfam database and as fCBD by SMART [101]. The constructed P. indica CBM1 model was further compared to all other CBM models from the Pfam database. LogoMat-P produced the best alignment for CBM1 and only small alignments of the HMMs for all other CBM domains indicating that the 69 domains identified in P. indica belong to the CBM1 category. (TIF) [file ppat.1002290.s011.tif]

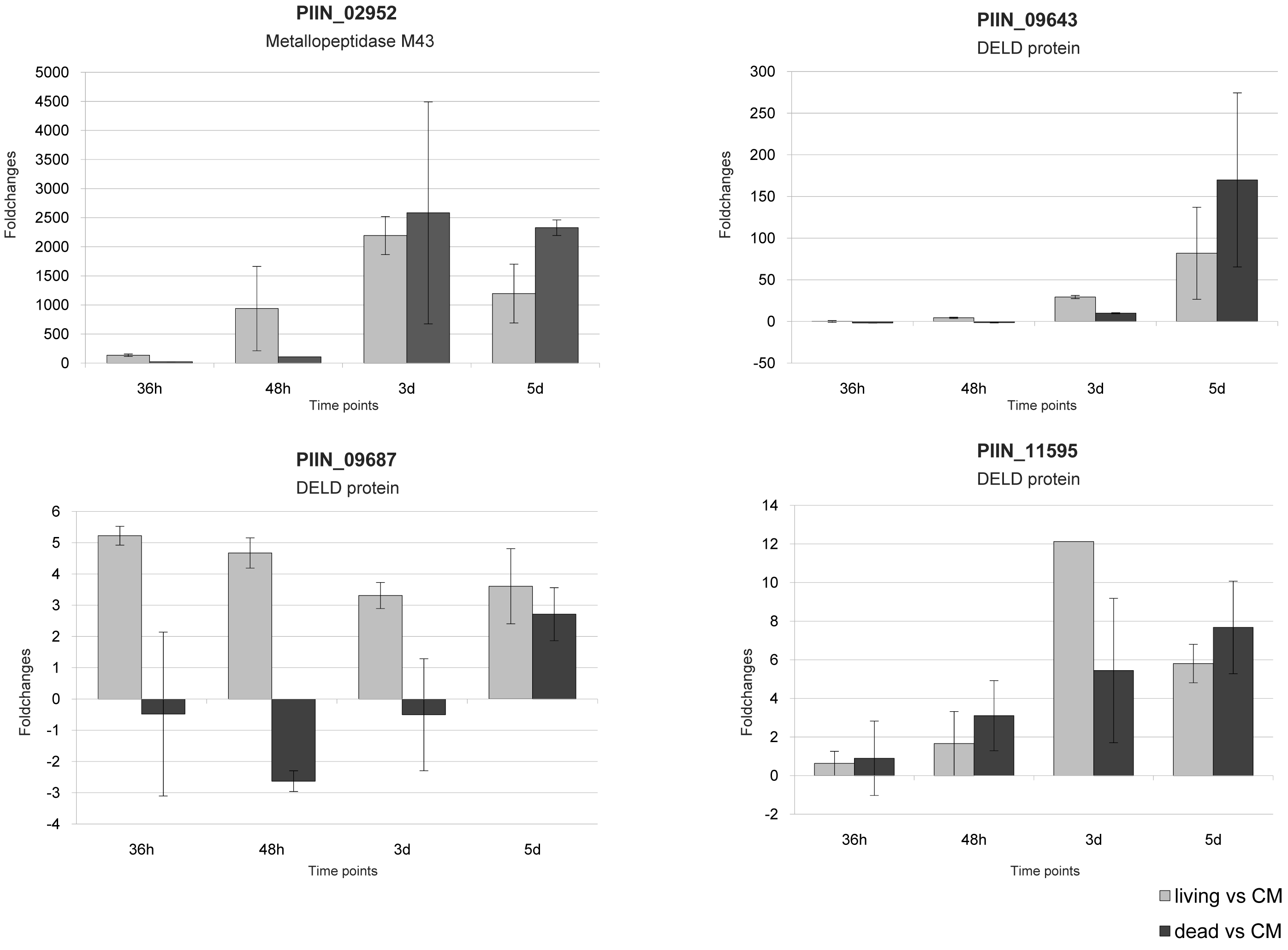

Supplement: Figure S12 — Mean fold-change estimates for selected P. indica genes for comparison between microarray and quantitative PCR methods. Fold changes were determined for P. indica growing on living (blue) or dead (red) barley roots by the 2−ΔCt method [118] and calculated relative to complete medium (CM) control. Expression data are standardized relative to PiTEF. Standard errors are from 3 independent biological repetitions. (TIF) [file ppat.1002290.s012.tif]

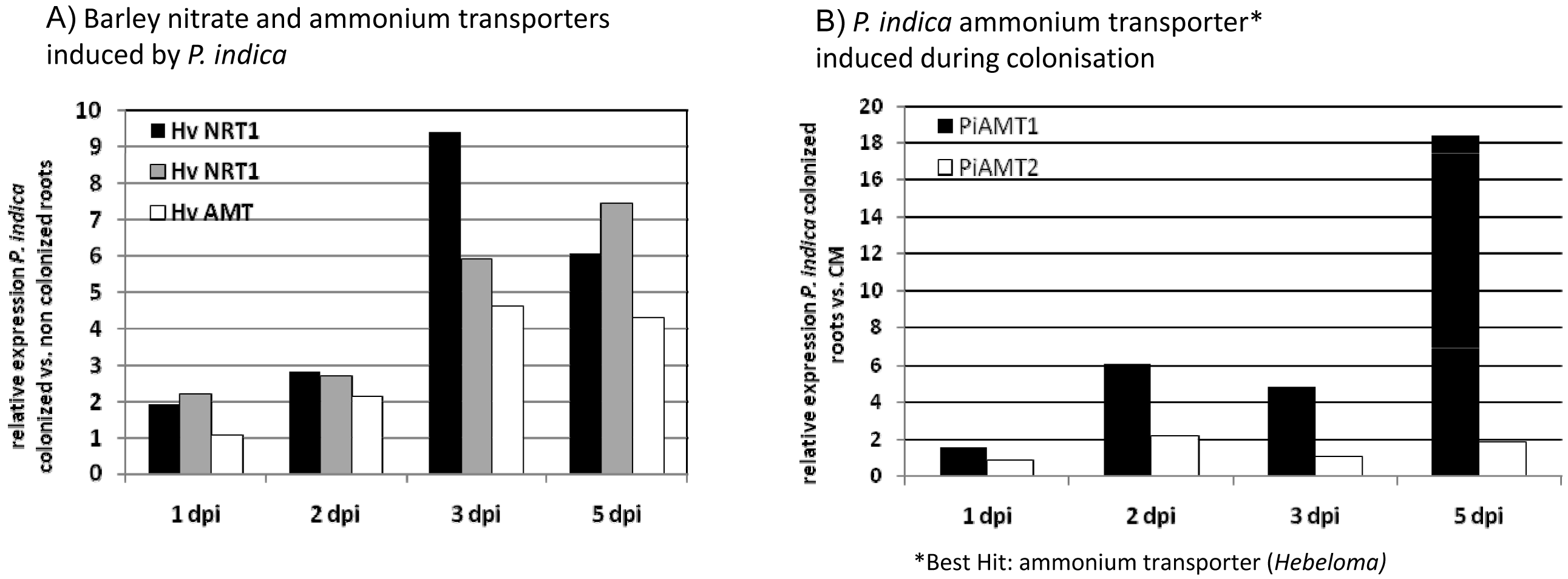

Supplement: Figure S13 — Quantitative PCR analysis of plant (left) and fungal (right) transporters involved in different forms of nitrogen uptake. The plant and fungal transporters were up-regulated upon P. indica colonization of barley roots grown on plant minimal medium (PNM) in axenic condition. A) Relative expression of two putative nitrate transporters (Hv NRT1, Harvest Unigene 46286 and 39899) and one ammonium transporter (Hv AMT, 10619) from barley in response to P. indica colonization at 1, 2, 3 and 5 dpi. Fold changes were determined by the 2−ΔCt method [118] and were calculated relative to non inoculated barley roots control. Expression data are calculated relative to barley ubiquitin (M60175). B) Relative expression of two P. indica ammonium transporters (PiAMT1, PIIN_02036; PiAMT2, PIIN_04373) during colonization of living barley roots. Fold changes were determined by the 2−ΔCt method and were calculated relative to P. indica TEF (AJ249911, PIIN_03008) versus the control complete medium. Relative expression values of selected transcripts were similar in 3 independent biological experiments. (TIF) [file ppat.1002290.s013.tif]

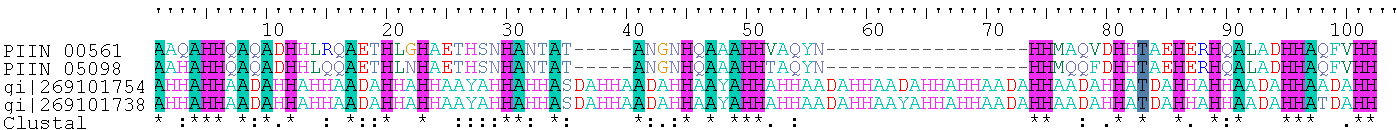

Supplement: Figure S14 — Alignment of the central part of Piriformospora indica DELD proteins and HRPII proteins from Plasmodium falciparum. Two representative DELD proteins and HRPII proteins were chosen for the alignment. While looking for DELD homologs from other organisms, we found that HRPII, a protein synthesized by the parasite during the early erythrocyte infection, shows about 30% sequence identity with the central part of the DELD proteins, primarily due to its high histidine and alanine content. (TIF) [file ppat.1002290.s014.tif]

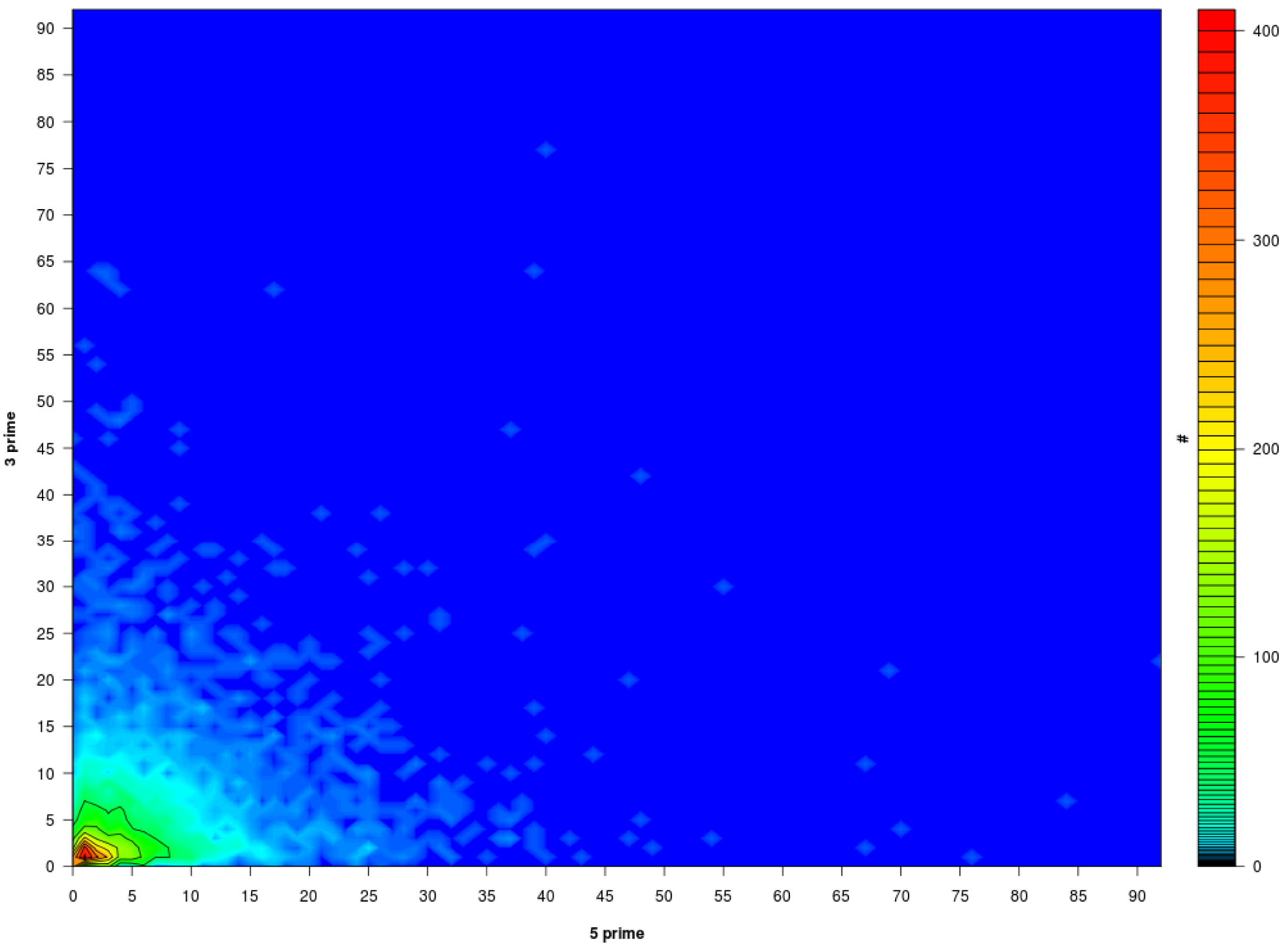

Supplement: Figure S15 — Distribution of P. indica intergenic region lengths. 9010 predicted genes were sorted into two dimensional bins on the basis of the lengths of the flanking intergenic distances to neighboring genes at the 5′ and 3′ ends as described before [119]. The number of genes in each bin is shown as a log transformed color-coded (z axis) heat map. 2759 genes either present alone or at the end of the scaffolds hence lacking neighboring genes were excluded from the analysis. P. indica genome does not show an unusual distribution of intergenic region lengths but possess a gene dense genome with an average distance between genes of 530 bp (see also Table S2). 279 genes were present in gene-poor regions with intergenic space between 1 kb and 9 kb. From the 279 genes, 43 (15.41%) were predicted to be secreted and of these, 18 (41.86%) were differentially regulated during colonization of barley roots. Additionally, similar to effectors found in other filamentous organisms, genes belonging to the P. indica putative effector family DELD proved to have flanking intergenic distances among the longest (with an average at the 5′ of 1677 bp and at the 3′ of 1345 bp). (TIF) [file ppat.1002290.s015.tif]

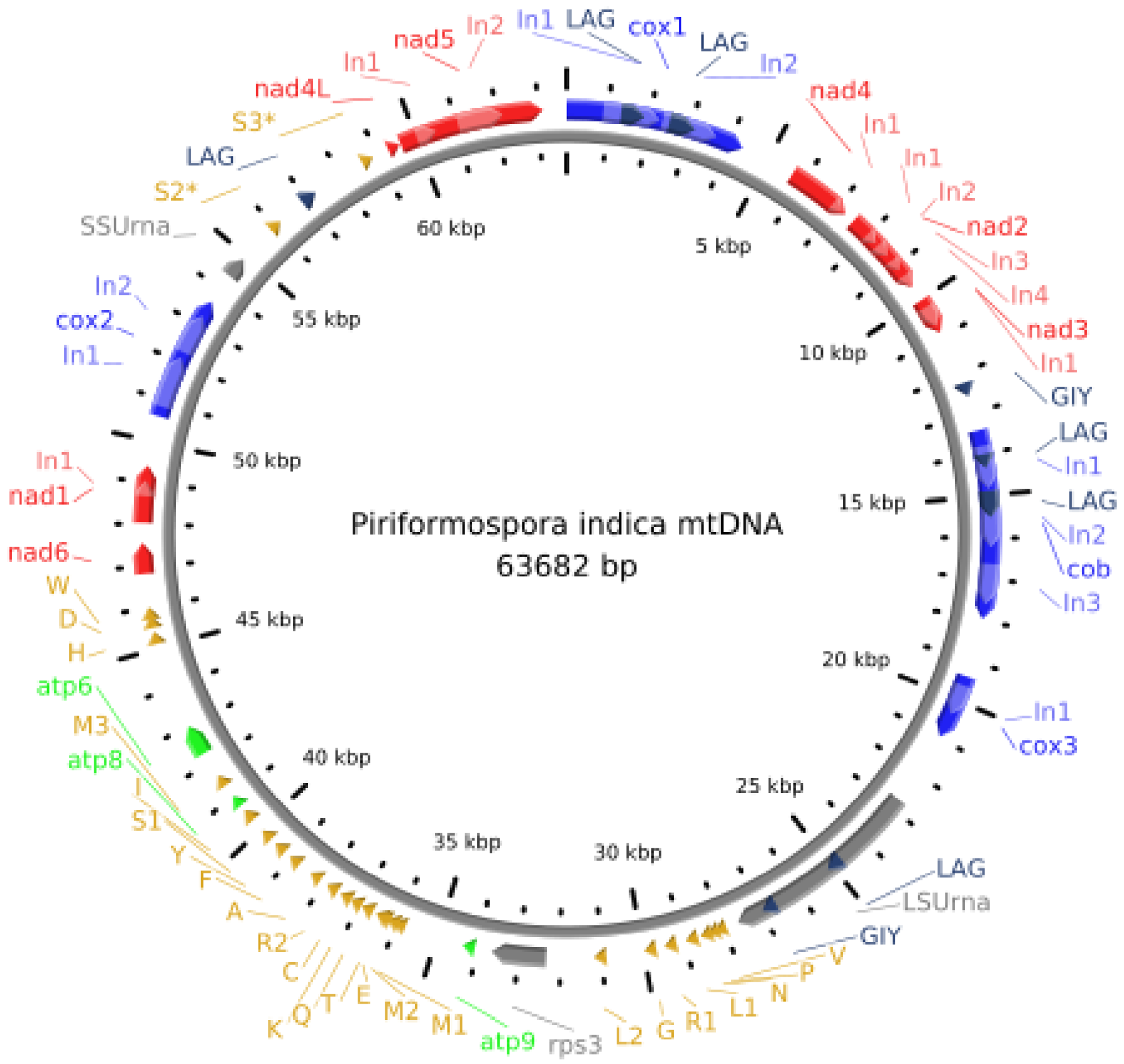

Supplement: Figure S16 — Circular map of the P. indica mitochondrion. The map was drawn using CGView [120]. Different colours indicate different gene families: blue: cytochrome c oxidase (subunits COX1, COX2, COX3) and cytochrome b (COB); red: NADH dehydrogenases (subunits NAD1, NAD2, NAD3, NAD4, NAD4L, NAD5, NAD6); green: ATPases (subunits ATP6, ATP8, ATP9); grey: subunits of the ribosome (SSUrna, LSUrna) and ribosomal proteins (rps3); gold: transfer RNAs (V = Valine, P = Proline, N = Asparagine, L = Leucine, R = Arginine, G = Glycine, M = Methionine, E = Glutamate, T = Threonine, Q = Glutamine, K = Lysine, C = Cysteine, A = Alanine, F = Phenylalanine, Y = Tyrosine, S = Serine, I = Isoleucine, H = Histidine, D = Aspartate, W = Tryptophan), numbers indicate multiple copies of the tRNA, asterisk indicate confirmation only by one program (tRNAscan-SE, [83], or Aragorn, [121]); dark blue: homing endonucleases with LAGLIDADG (LAG) or GIY-YIG (GIY) motif; introns are drawn in the same colour as their corresponding genes, but a bit lighter; numbers indicate the position and number of introns in the specific gene (In1–In4). (TIF) [file ppat.1002290.s016.tif]
